# Hyper Cross-linked Poly(ether imide) via Friedel–Crafts Acylation and Dehydrochlorination

**DOI:** 10.1021/acs.macromol.5c00532

**Published:** 2025-06-25

**Authors:** Connor S. Thompson, Carlos Posada, Guoliang Liu

**Affiliations:** † Department of Chemistry, 1757 Virginia Tech, Blacksburg, Virginia 24061, United States; ‡ Macromolecules Innovation Institute, Virginia Tech, Blacksburg, Virginia 24061, United States; § Division of Nanoscience, Academy of Integrated Science, Virginia Tech, Blacksburg, Virginia 24061, United States; ∥ Department of Chemical Engineering, Department of Materials Science and Engineering, Virginia Tech, Blacksburg, Virginia 24061, United States

## Abstract

Poly­(ether imide) (PEI), a high-performance thermoplastic, is known for its high mechanical strength, thermal stability, and great processability. PEI is solution-processable because it is soluble in chloroform, dichloromethane, dimethylformamide, tetrahydrofuran, and *N*-methyl pyrrolidone, unlike other high-performance thermoplastics. However, this behavior limits its applications because potential exposure to these solvents can cause structural instability. Herein, we report a method to counteract this drawback by preparing a thermally cross-linked PEI through postpolymerization modification of the polymer backbone. Via Friedel–Crafts acylation with a thermally active moiety of 3-chloropropionyl chloride (3-CPC), the resulting P­(EI-CPC_*x*_) retains solution processability and is used as an additive in virgin PEI, generating thermally stable and solvent-resistant PEI upon thermal annealing-induced dehydrochlorination and cross-linking. Dynamic mechanical analysis and swelling tests corroborate these findings with storage modulus, glass transition, and swelling ratios, which correlate positively with increasing cross-link density. This work advances engineering polymer chemistry and opens new routes for postpolymerization modification of high-performance thermoplastics.

## Graphical abstract

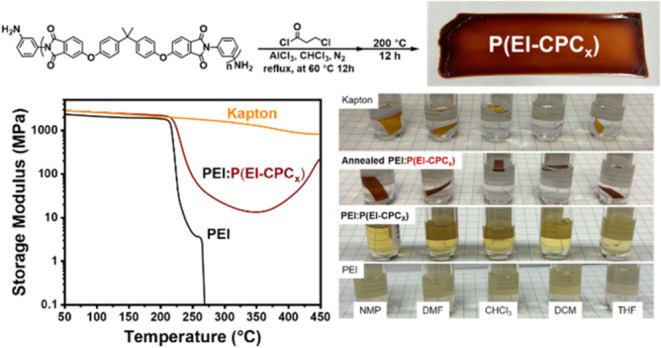

## Introduction

Poly­(ether imide) (PEI) is an attractive high-temperature engineering thermoplastic with excellent thermal properties, mechanical strength, and processability thanks to the flexible isopropylidene group and ether linkages.^1–3^ These flexible linkages decrease the melt-processing temperature of PEI to ∼260–370 °C, compared to ∼350–400 °C for other engineering thermoplastics such as poly­(ether ether ketone) (PEEK).^4^ In addition, unlike PEEK and other polyimides, PEI is soluble in solvents such as *N*-methyl pyrrolidone (NMP), *N*,*N*-dimethylformamide (DMF), dichloromethane (DCM), chloroform (CHCl_3_), and tetrahydrofuran (THF).^5^ This inherent solubility is appealing because it leads to great solution processability, but also represents a major drawback of PEI, keeping it from replacing Kapton and PEEK in applications that demand high solvent resistance and robustness.^6–9^ To rectify this drawback, novel chemistry is needed to synthesize solvent-resistant PEIs.

High-performance thermoplastics such as PEEK and Kapton are known for their superior solvent resistance.^10^ Due to this inherent solvent resistance, it is challenging to modify these polymers via postpolymerization modification. Even when they could be modified, it often leads to undesirable outcomes. For instance, sulfonation can functionalize PEEK into ionomers but, in turn, decreases the solvent resistance.^11,12^ Alternatively, high-performance engineering plastics were functionalized via monomer modification, end-group functionalization, and other methods.^13–16^ In particular, PEI was functionalized at the end groups using phosphonium,^17,18^ sulfonate salts,^19,20^ and ureidopyrimidinone,^21^ resulting in enhanced thermal and mechanical properties. However, these methods could not covalently cross-link PEI, thus offering little or no solvent resistance. Covalent “crosslinking” of PEI could be achieved via ultraviolet light (UV) irradiation or thermal cross-linking.^22^ Because PEI highly absorbs UV light, the destructive UV irradiation degraded the polymer backbone and its properties.^23^ Therefore, thermal cross-linking is preferable due to its less destructive nature.^14,24^ Previously, our group used an end-group modification approach to synthesize PEI with azide terminal groups.^14^ Upon heating, the azide-functionalized PEI cross-linked to form a thermoset. The PEI cross-linking via azide decomposition was effective, but the PEI chain length limited the cross-linking density. This limited degree of functionalization is true for all end-group modifications, as there is an inversely proportional relationship between molar mass and the number of end groups.^25^ Therefore, we postulate that backbone functionalization is advantageous in increasing the polymer cross-linking density compared to end-group functionalization.

Recent reports outlined the use of Friedel–Crafts acylation to modify aromatic thermoplastics,^15,26–28^ where an acyl halide was treated with a Lewis acid to produce a carbocation electrophile for aromatic substitution in the polymer backbone.^29^ Ferreira et al. demonstrated that the aromatic backbone in PEI can undergo acylation (up to 55%) with acetyl, butanoyl, hexanoyl, decanoyl, and benzoyl chlorides in the presence of AlCl_3_.^27,28^ Upon acylation, these functionalized aromatic thermoplastics supposedly could cross-link to produce materials with high solvent resistance. Thus, we hypothesize that inserting reactive moieties along the PEI chains can effectively cross-link PEI at higher densities than end-group functionalization. Different from most cross-linking chemistries, PEI cross-linking could be achieved through dehydrochlorination, similar to the thermal dehydrochlorination of poly­(vinyl chloride) (PVC), which generates a highly reactive environment leading to cross-linking.^30^ To introduce dehydrochlorination moieties, we selected 3-chloropropionyl chloride (3-CPC) containing an acyl chloride and an alkyl chloride, where the latter could undergo dehydrochlorination upon thermal annealing.

In this work, we have synthesized a series of backbone functionalized PEIs via Friedel–Crafts acylation with 3-CPC. These functional PEIs were solution-mixed with virgin PEI and cast into films. Thermal annealing dehydrochlorinated the attached 3-CPC moieties and cross-linked the PEIs. These cross-linked PEIs exhibited enhanced thermomechanical properties and, in contrast with traditional PEI, high solvent resistance to NMP, DMF, CHCl_3_, DCM, and THF, akin to other high-performance thermoplastics such as Kapton.

## Experimental Section

### Materials

3-Chloropropionyl chloride (3-CPC, 90% purity), aluminum chloride, poly­(ether imide) (weight-average molar mass, *M*_w_ = 28 kDa), and *N*-methyl pyrrolidone (NMP, Reagentplus 99%) were received from Sigma-Aldrich and used as received. 3-CPC was kept in a freezer before use. Dichloromethane (DCM), chloroform (HPLC grade), tetrahydrofuran (THF), and *N*-methyl formamide (DMF, HPLC grade) were received from Fisher Chemical. Acetone (analytical pure, Acros Organics) and isopropanol (VWR chemicals) were all used as received.

### Synthesis of P­(EI-CPC_*x*_)

PEI was functionalized with varying equivalents of acyl chloride, namely 3-CPC, via Friedel–Crafts acylation using AlCl_3_, following previous work by Dr. Conceição and co-workers^27,28^ with slight modifications. Typically, 1 g of PEI was dissolved in 15 mL of CHCl_3_ in a two-neck round-bottom flask. After adding AlCl_3_, the flask was purged with N_2_, sealed, and stirred at 0 °C. A solution of 3-CPC dissolved in CHCl_3_ (5–15% by volume) was added dropwise into the flask while stirring vigorously. The solution was then heated to 60 °C and refluxed for 12 h. The vessel was cooled to 0 °C. Unreacted AlCl_3_ and 3-CPC were quenched by adding 30 mL of deionized (DI) water. After stirring for 1 h, the solution was centrifuged at 10,000 rpm for 10 min. The top aqueous layer was decanted. The process of quenching, centrifugation, and decanting was repeated three times to ensure complete removal of AlCl_3_. The bottom organic layer was passed through sodium sulfate and crashed out dropwise into a mixture of acetone and IPA (25:75 by volume). The mixture was stirred for 12 h and separated via vacuum filtration. Finally, the resulting P­(EI-CPC_*x*_) polymer powder, where *x* denotes the number of CPC units per PEI monomer, was dried at 80 °C for 12 h before further characterization.

### Thin Film Preparation

P­(EI-CPC_*x*_) was mixed with PEI at various mass ratios (P­(EI-CPC_*x*_)/PEI = 3:7, 5:5, and 7:3) and dissolved in CHCl_3_ to reach a final concentration of 100 mg polymer per 1 mL CHCl_3_. The solutions were cast on glass slides and air-dried for 12 h, resulting in free-standing films. The films showed no signs of haziness, and small-angle laser light scattering of the films displayed no scattering halos, suggesting full miscibility between PEI and P­(EI-CPC_*x*_) (Figure S1).^31^ The solution-cast films were thermally annealed and cross-linked in a temperature-programmable vacuum oven. Starting at room temperature, the oven was ramped to 100 °C and held for 6 h to ensure solvent removal. Afterward, the oven was ramped to 160 °C at a rate of 10 °C/h and then to 220 °C with a reduced rate of 5 °C/h to prevent film foaming. The oven was held at 220 °C for 6 h to ensure sufficient dehydrochlorination, releasing HCl gas (Figure S2). The resultant cross-linked films were cooled to RT.

### Cross-linking Density Evaluation

Following our previous work,^14^ polymer cross-linking density (*v*) and average molar mass between cross-links were estimated using the equilibrium elastic modulus at 300 °C with eq 1.

1 $$G^{\prime }=\frac{E^{\prime }}{2\left(1+\sigma \right)}=\mathrm{RT}\frac{\rho }{M_{c}}=\mathrm{RTv}$$

where *G*′ is the shear modulus at equilibrium, *E*′ is the tensile modulus, σ is the Poisson ratio, and ρ is the density of the polymer network. For simplicity, the Poisson ratio of P­(EI-CPC_*x*_) was assumed to be the same as that of PEI (σ = 0.36, the standard value for Ultem). *E*′ of each composite was measured at 300 °C using DMA.

### Swelling Testing

Solvent resistances were evaluated by submerging polymer films (10 mm × 3 mm in size) in various organic solvents (CHCl_3_, NMP, DCM, DMF, and THF) over 7 days. To determine the swelling ratio, the area of the films was measured before and after soaking (Figure S3), following a previously reported procedure.^32,33^ Swelling ratios were then determined using eq 2

2 $$\mathrm{SR}\left(\%\right)=\frac{A_{\mathrm{wet}}-A_{\mathrm{dry}}}{A_{\mathrm{dry}}}\times 100$$

where *A*_dry_ is the area of the film before soaking, and *A*_wet_ is the area of the film after soaking for 7 days.

### Characterization

Proton nuclear magnetic resonance (^1^H NMR) spectroscopy was performed using a Bruker Avance III 600 at 599.98 MHz in CDCl_3_. Fourier transform infrared spectroscopy (FTIR) was performed using a PerkinElmer ATR-FTIR (model Spectrum 100) at room temperature in the range of 4000–500 cm^–1^ (128 scans and 4 cm^–1^ resolution). Differential scanning calorimetry (DSC) was performed using Q2500 Discovery (TA Instruments). To conduct the DSC measurements, polymers (∼5–10 mg) were preheated at 80 °C for 12 h to remove residual solvents and then added to hermetic pans. Samples were then subjected to a 10 °C/min temperature ramp from 25 to 280 °C, and the heat flow was analyzed. Thermogravimetric analysis (TGA) was performed as follows. After predrying the polymers at 80 °C for 12 h, the polymers were subjected to thermogravimetric analysis (TGA5500 Discovery, TA Instruments) by ramping from 50 to 900 °C (ramp rate, 10 °C/min) under a nitrogen flow (40 mL/min). Dynamic mechanical thermal analysis (DMTA) was performed using a DMA Q800 (TA Instruments) fitted with tension clamps. Films were loaded into tension clamps with a torque of 3 N and a preload force of 0.01 N. Storage modulus (tensile modulus, *E*′) and tan δ were obtained utilizing DMTA over a temperature range of 25–450 °C (ramp rate, 3 °C/min; frequency, 1 Hz) and a maximum strain of 125%. Small-angle laser light scattering was performed using a laser font of 3 mW He–Ne laser (λ = 632.8 nm), and an AmScope digital camera (MU503B) was used to acquire the scattering patterns (*V*_v_ mode, 24 cm). Phase contrast optical microscopy was performed using a Nikon Eclipse LV100 equipped with an AmScope digital camera (MU503B) (phase contrast mode with a 20× Ph1 objective). Energy Dispersive X-ray Spectroscopy (EDS) was performed using a JEOL IT-500HR (working distance of 10 mm at 10 kV).

## Results and Discussion

### Synthesis of P­(EI-CPC_*x*_)

PEI was functionalized at the backbone using Friedel–Crafts acylation. Because the degree of functionalization depended on the equivalence of reactants,^27,28^ we explored the effect of changing the equivalence, i.e., the molar ratio among AlCl_3_, 3-CPC, and PEI, in the range of 3:3:1 to 6:6:1 (Figure 1 and Table 1). Once PEI was functionalized by 3-CPC, the P­(EI-CPC_*x*_) and virgin PEI were characterized using ^1^H NMR (Figure 2). For virgin PEI, each proton peak was assigned a number, in corroboration with a previous report.^28^ All aromatic peaks were in the range of 7.0–8.0 ppm, and the aliphatic protons at 1.77 ppm (**6**). Imide protons **1**, **2**, and **3** appeared at 7.90, 7.37, and 7.44 ppm, respectively.^28^ Aromatic protons on the bisphenol A (BPA) moiety appeared at 7.05 ppm (**4**) and 7.35 ppm (**5**). The aryl group (phenyl) protons **7**, **8**, and **9** were at 7.51, 7.61, and 7.66 ppm, respectively. Triplet peaks at 3.95 and 3.47 ppm of the functionalized P­(EI-CPC_*x*_) were associated with the attached moiety from 3-CPC. For P­(EI-CPC_0.06_) produced with three equivalents of AlCl_3_ and 3-CPC with respect to PEI (i.e., AlCl_3_/3-CPC/PEI = 3:3:1), the resonance peaks of the aromatic protons (**1–5**, **7–9**) did not shift significantly. At higher equivalents (AlCl_3_/3-CPC/PEI = 4:4:1 or more), the aromatic protons on the imide (**1–3**), BPA (**4** and **5**), and phenyl (**7**-**9**) groups exhibited major reductions in intensities and downfield shifting (Table S1). In addition to the drastic effects on the aromatic protons, a decrease in intensity and a shift of the peak position (from 1.77 to 2.45 ppm) associated with the methyl of the polymer backbone were detected with increasing functionalization.

**Figure 1:**
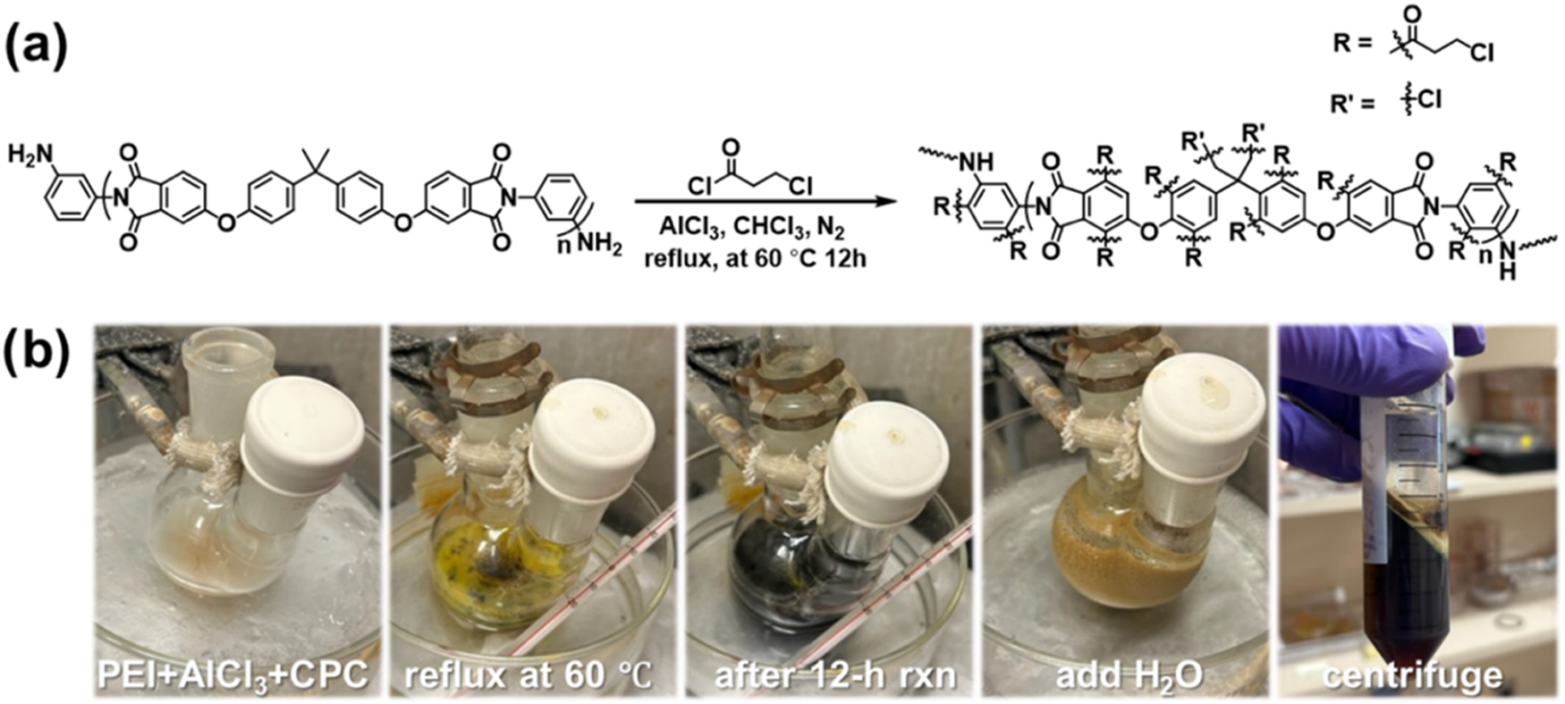
Functionalization of PEI with 3-CPC. (a) Friedel–Crafts acylation of PEI using 3-CPC. R shows the potential sites for functionalization. (b) Photographs of the reaction at various stages.

**Figure 2:**
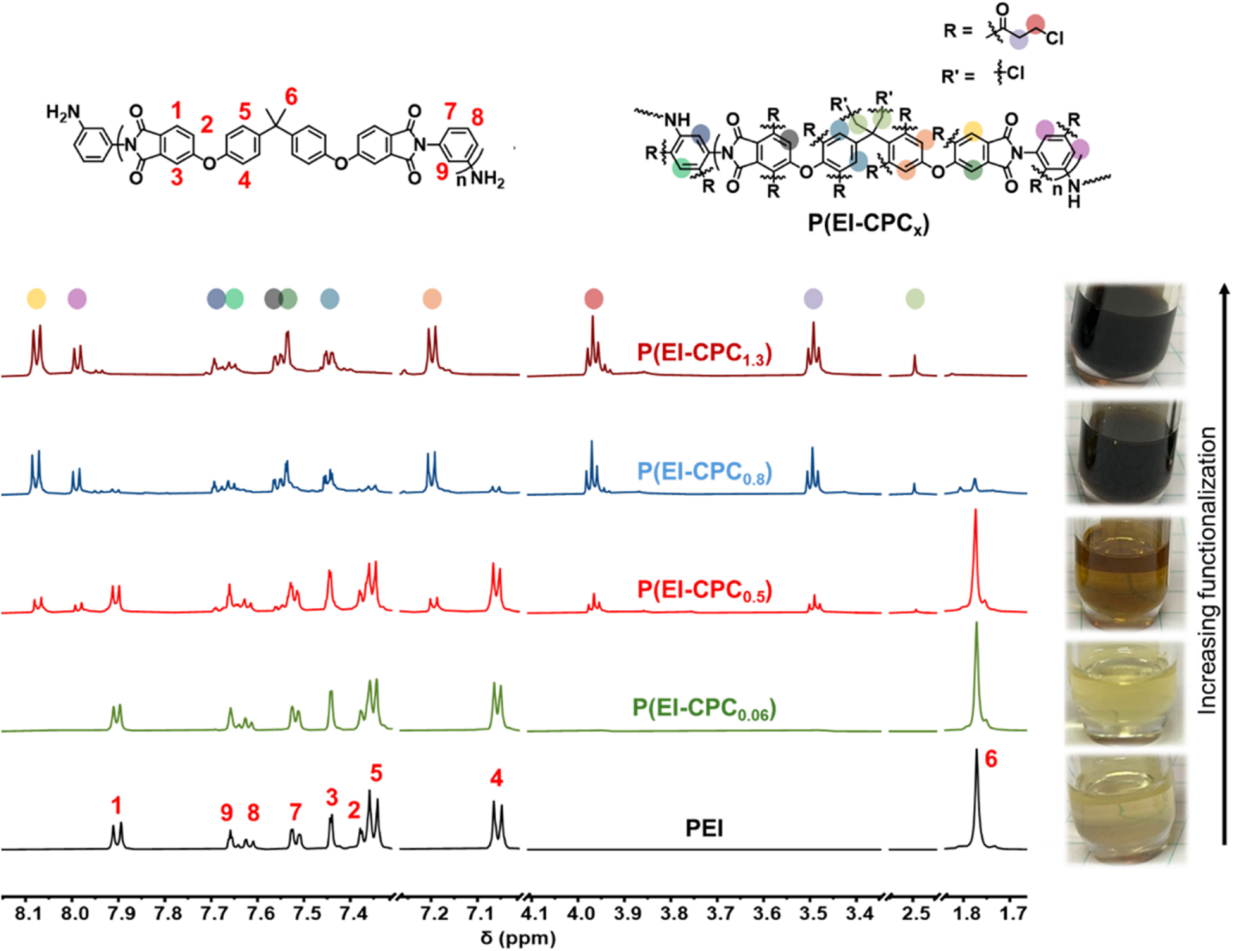
^1^H NMR of virgin PEI and P­(EI-CPC_*x*_) derivatives. Characteristic protons in virgin PEI and P­(EI-CPC_*x*_) are highlighted by numbers (**1**–**9**) and colors, respectively.

**Table 1:** Synthesis Condition and Properties of P­(EI-CPC_*x*_)

	feed ratio^a^	CPC/PEI^b^	*T*_g_ (°C) (DSC)	*T*_d_ (°C) (TGA)	casting behavior
P(EI-CPC_1.3_)	6:6:1	1.27	n/a	511	brittle
P(EI-CPC_0.8_)	5:5:1	0.83	n/a	518	brittle
P(EI-CPC_0.5_)	4:4:1	0.54	220	536	brittle
P(EI-CPC_0.06_)	3:3:1	0.058	218	539	coherent
PEI	0	0	217	554	coherent

a Molar feed ratio of AlCl_3_/3-CPC/PEI.

b Average number of 3-CPC units per PEI monomer.

The degree of functionalization was determined via ^1^H NMR using an internal standard of dichloromethane (Figures S4–S7 and Table 1). At the lowest equivalence (AlCl_3_/3-CPC/PEI = 3:3:1), the number of moieties of 3-CPC was calculated to be ∼0.06 per ether imide (EI) monomer. When the equivalence was increased to AlCl_3_/3-CPC/PEI = 4:4:1, the number of 3-CPC moieties per PEI monomer increased to ∼0.5. At higher equivalences of AlCl_3_/3-CPC/PEI = 5:5:1 and 6:6:1, each PEI repeating unit contained ∼0.8 and ∼1.3 moieties of 3-CPC, respectively, establishing a linear relationship between the amount of equivalence used and the degree of functionalization (Figure S8).

The shifts of the imide, BPA, and phenyl protons are caused by the insertion of a carbonyl group from 3-CPC. Carbonyls are known electron-withdrawing groups, which effectively deshield the aromatic protons, causing a downfield shift in ^1^H NMR.^34^ This downfield shift was observed in all aromatic protons on the backbone of P­(EI-CPC_*x*_). Previously, Conceição and co-workers reported no functionalization at the phenyl (**7–9**) or methyl (**6**) groups of PEI.^27^ In addition, they reported a degree of functionalization in the range of 0.04–0.55 acyl chloride moieties per monomer. In contrast, our P­(EI-CPC_*x*_) showed functionalization not only at the BPA and imide moieties but also at the phenyl, and the degrees of functionalization ranged from 0.06 to 1.3 acyl chloride moieties per monomer. The methyl groups were also partially halogenated after the reaction. These differences are likely caused by the use of 3-CPC and the abundant HCl production in comparison to Conceição and co-workers.^27,28^ Under our reaction conditions, a significant excess of Lewis acid (6 equiv to PEI) was used, which induced the Friedel–Crafts reaction of 3-CPC with PEI, producing a large quantity of HCl and resulting in a highly acidic environment. Compared to the reactions by Conceição and co-workers,^27,28^ HCl in our reactions was not removed and it potentially interacted with the excess AlCl_3_ to form AlCl_3_–HCl, a known “superacid” with an acidity greater than 100% sulfuric acid (Scheme S1).^35–37^ This “superacid” likely deprotonated the methyl groups in PEI, via a potential pathway of initial protonation followed by hydrogen gas release,^38^ leaving behind a carbocation for halogenation (Scheme S1). This process would promote chlorine substitution at the methyl groups, which could explain the decreasing intensity at 1.77 ppm and the appearance of a new peak at 2.45 ppm.

Besides ^1^H NMR, FTIR spectra of P­(EI-CPC_*x*_) exhibited characteristic changes after PEI functionalization (Figure 3). In virgin PEI, aromatic alkene C–H stretching was prominent at 1013 cm^–1^, but its intensity decreased after functionalization with 3-CPC. Upon attaching the 3-CPC moiety to PEI, the aromatic C–H bonds were cleaved, producing HCl and a new C–C bond between PEI and the 3-CPC moiety. The attached 3-CPC moiety brought about a new carbonyl absorption band at 1684 cm^–1^, which appeared as a shoulder peak alongside the typical carbonyl stretching at 1719 cm^–1^ associated with the backbone imide.^39^ The imide carbonyl was at a higher frequency because of the neighboring strongly electron-withdrawing nitrogen, whereas the carbonyl from the 3-CPC moiety contained an alkyl halide, which is weakly electron-withdrawing, thus it appeared at a slightly lower frequency.^40^ The attachment of the 3-CPC moiety to PEI also yielded a new C–Cl stretching peak at 561 cm^–1^. These changes in C=O, C–H, and C–Cl peak intensities confirmed the functionalization of PEI with 3-CPC, corroborating the observations from ^1^H NMR.

**Figure 3:**
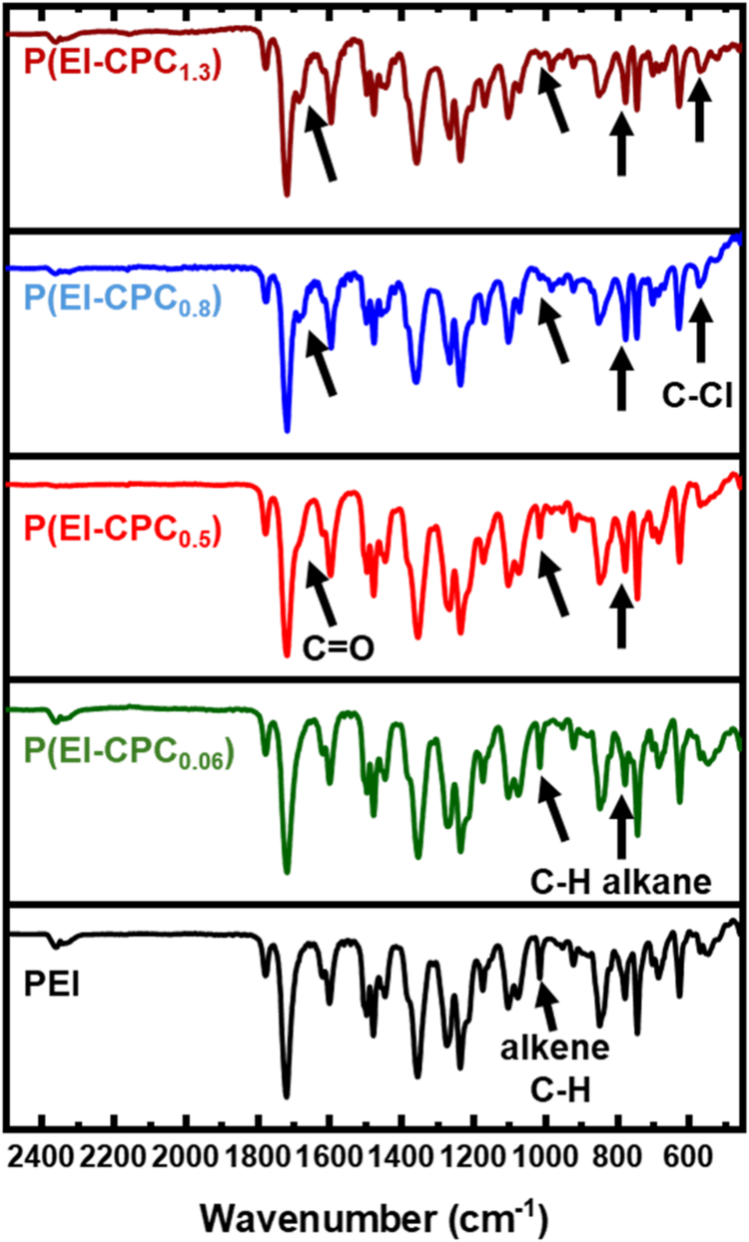
FTIR of functionalized P­(EI-CPC_*x*_) and PEI. The carbonyl peak increased with an increasing degree of functionalization.

### Thermal and Mechanical Properties of P­(EI-CPC_*x*_)

Differential scanning calorimetry (DSC) was employed to evaluate the thermal properties of functionalized P­(EI-CPC_*x*_). DSC traces of virgin PEI showed a single thermal event at 217 °C, corresponding to its glass transition temperature (*T*_g_) (Figure 4). However, P­(EI-CPC_*x*_) displayed a new broad thermal event in a variable range depending on the level of functionalization. For instance, the thermal event of P­(EI-CPC_0.06_) began at ∼150 °C and persisted until ∼215 °C, then underwent a glass transition similar to virgin PEI (Figure 4, green trace) (Table 1). As the degree of functionalization increased, the thermal event became increasingly broad and apparent. In contrast, this thermal event of P­(EI-CPC_0.5_) began at ∼150 °C and persisted to 221 °C, and yet no *T*_g_ was detected. For P­(EI-CPC_0.8_) and P­(EI-CPC_1.3_), this phenomenon was further pronounced. Their thermal events broadened to the range of ∼120–200 °C. Once the PEI-CPC underwent the first heating ramp, a second DSC heating ramp was applied. P­(EI-CPC_0.06_) and P­(EI-CPC_0.5_) exhibited *T*_g_ values at 218 and 220 °C, respectively (Figure 4 and Table 1). However, P­(EI-CPC_0.8_) and P­(EI-CPC_1.3_) displayed no thermal events or glass transitions.

**Figure 4:**
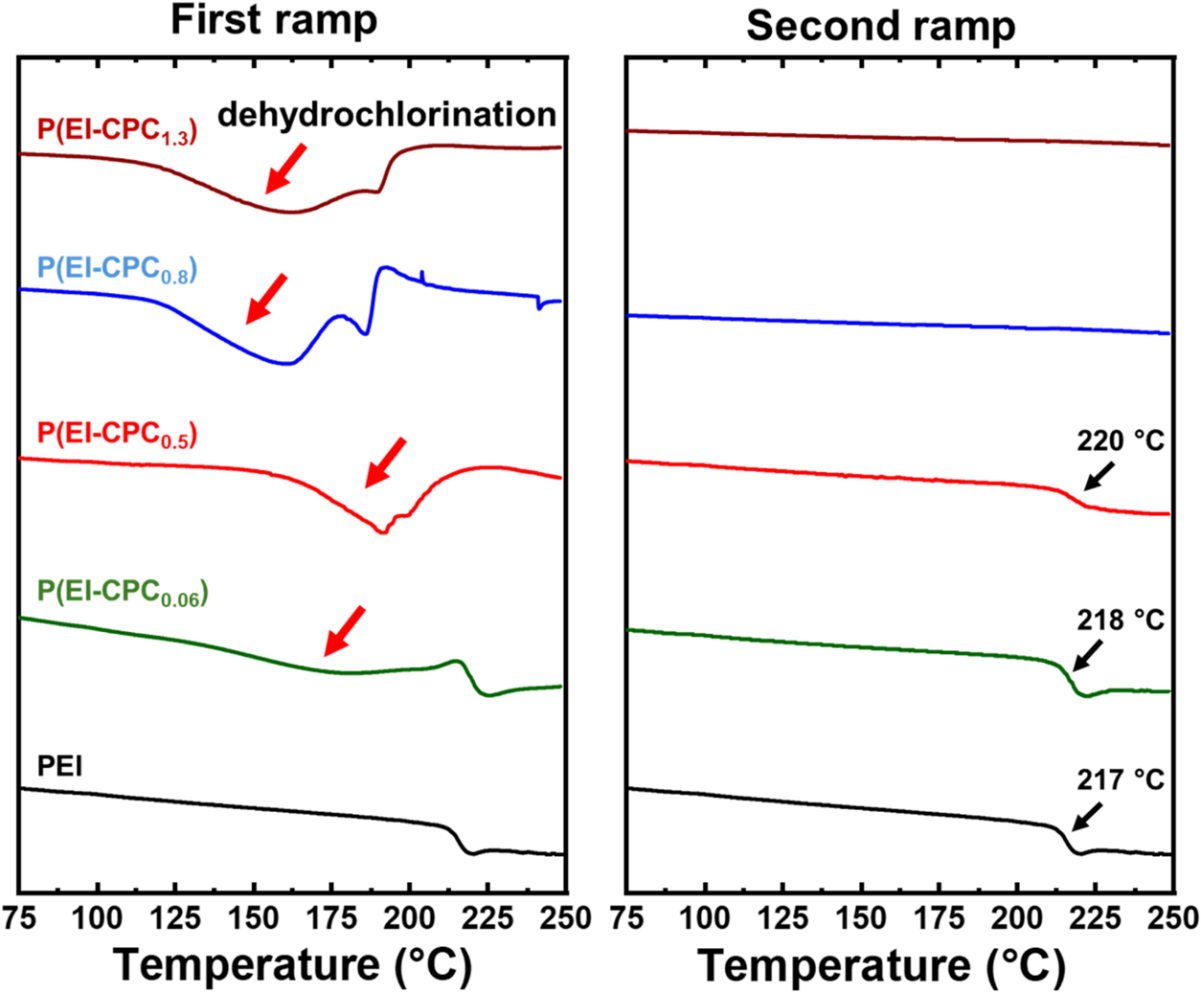
DSC traces of P­(EI-CPC_*x*_) in comparison to virgin PEI. (left) The first ramp illustrated the dehydrochlorination reaction and (right) the second ramp after the initial heating.

As the degree of functionalization in P­(EI-CPC_*x*_) increased, the thermal event onset at lower temperatures and covered a broader range. The thermal event coincided with the low-temperature dehydrochlorination of PVC,^41^ thus attributed to the dehydrochlorination of the attached CPC moieties. However, dehydrochlorination occurred at different temperatures depending on the degree of functionalization in P­(EI-CPC_*x*_). Ferreira et al. reported that *T*_g_ of PEI decreased as the degree of functionalization increased, as the attached side chain decreased the number of interchain interactions.^27^ In our functionalized P­(EI-CPC_*x*_), *T*_g_ likely decreased as well, albeit not detectable within the first heating ramp due to the overlap with dehydrochlorination. The functional moieties enhanced the mobility of PEI chains, allowing for dehydrochlorination to occur at lower temperatures.

Dehydrochlorination was completed within the first heating ramp, and the second heating ramp showed no signs of dehydrochlorination. Instead, P­(EI-CPC_0.06_) and P­(EI-CPC_0.5_) exhibited a slightly increased *T*_g_, indicative of potential cross-linking. Supposedly, thermally treating the 3-CPC moieties created highly reactive intermediates for cross-linking, similar to the cross-linking of PVC upon heating (Scheme S2).^30^ P­(EI-CPC_0.8_) and P­(EI-CPC_1.3_) underwent no visible glass transition, probably because the polymer chains were highly cross-linked and no longer partook in long-range segmental motions.^42,43^ The extent of restriction to the segmental motions appeared to be dependent on the degree of functionalization.

The degradation of functionalized P­(EI-CPC_*x*_) was assessed using thermogravimetric analysis (TGA) and compared with that of virgin PEI. To remove the potential interference by dehydrochlorination and any residual solvents, all P­(EI-CPC_*x*_) were subject to thermal annealing before TGA. After thermal annealing, virgin PEI degraded at ∼550 °C, whereas P­(EI-CPC_0.06_) degraded at ∼540 °C (Figure 5) (Table 1). P­(EI-CPC_*x*_) showed lower *T*_d_ values than virgin PEI. As the degree of functionalization increased, the thermal degradation temperature (*T*_d_) of P­(EI-CPC_*x*_) decreased. P­(EI-CPC_0.5_), P­(EI-CPC_0.8_), and P­(EI-CPC_1.3_) showed *T*_d_ values of ∼535, 520, and 510 °C, respectively. It is reported that functionalized and cross-linked polyimides typically have decreased *T*_d_ due to an increasing amount of heteroatoms and conjugation.^14,44–46^ In this work, the lower *T*_d_ of P­(EI-CPC_*x*_) can also be attributed to functionalization and cross-linking. Upon dehydrochlorination, P­(EI-CPC_*x*_) supposedly cross-links through the 3-CPC moiety, forming covalent C–C bonds (Scheme S2). The oxygen heteroatoms from the 3-CPC moiety induce a decreased *T*_d_. This early onset of degradation, however, does not compromise the mechanical properties of PEI at its common operating temperatures (<300 °C), similar to our previous work.^14^

**Figure 5:**
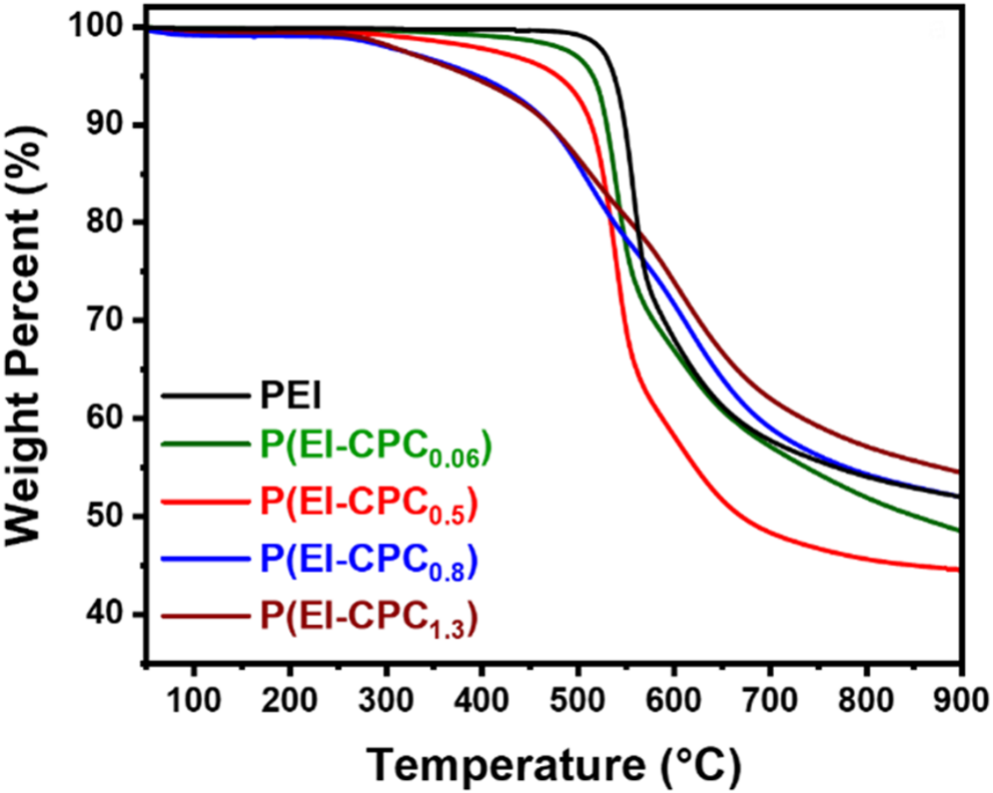
TGA of P­(EI-CPC_*x*_) compared to virgin PEI after an initial annealing step.

In addition to the thermal properties, the film-casting behaviors of P­(EI-CPC_*x*_) appeared to be significantly different from those of virgin PEI. Solution-cast films of P­(EI-CPC_*x*_) became increasingly brittle as the degree of functionalization was increased (Figure 6). After additional thermal annealing at 220 °C, these films demonstrated various levels of solvent resistance to CHCl_3_ (Figure 6). P­(EI-CPC_0.06_) displayed minor solvent resistance and was partially dissolved. P­(EI-CPC_0.5_) was partially swollen. P­(EI-CPC_0.8_) and P­(EI-CPC_1.3_) remained intact in CHCl_3_ and showed no signs of dissolution or swelling, suggestive of polymer cross-linking.^14^ The brittle casting behavior of the P­(EI-CPC_*x*_) was caused by the increasing number of 3-CPC moieties on the PEI backbone, which possibly caused a loss of secondary relaxations, leading to film embrittlement.^47–49^ This effect was similar to the PEI films with a degree of functionalization of 0.55 in the work by Ferreira et al.^27^ In this work, P­(EI-CPC_*x*_) with a degree of functionalization of *x* ≥ 0.54 showed the same behavior.

**Figure 6:**
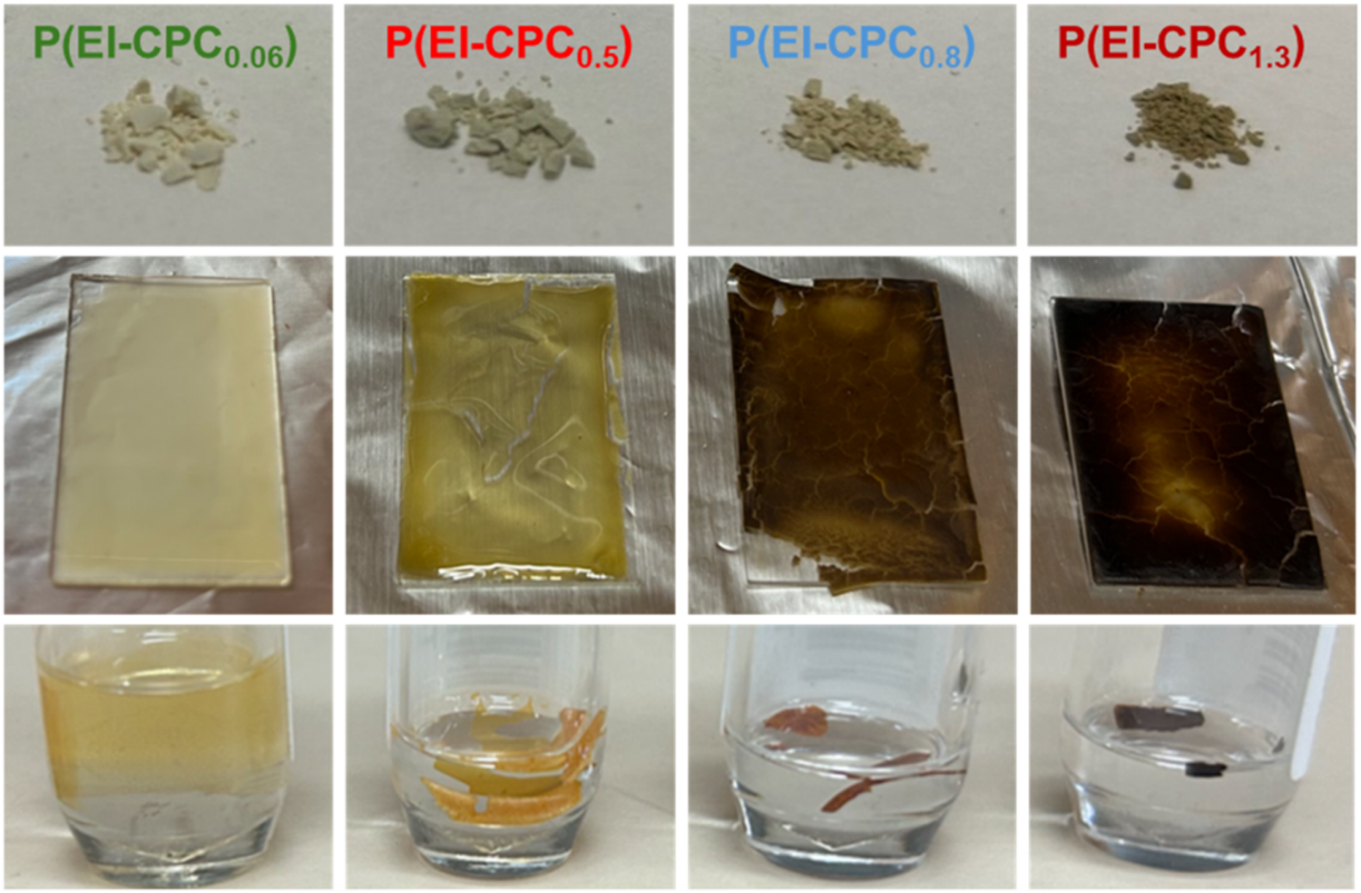
Images of P­(EI-CPC_*x*_) in the form of powder (top), thin film (middle), and immersion in CHCl_3_ after thermal annealing (bottom).

### Thin film of PEI/P­(EI-CPC_*x*_) mixtures

Because P­(EI-CPC_*x*_) could not be easily cast into coherent films, we considered mixtures of P­(EI-CPC_*x*_) and virgin PEI to improve film formability. The weight mixing ratio (ϕ) of PEI/P­(EI-CPC_*x*_) was varied from 70:30 to 50:50 and 30:70. Films of PEI/P­(EI-CPC_0.06_) of all mixing ratios were intact after solution-casting (Figure 7), and their color darkened after thermal annealing (Figure 7 insets). PEI/P­(EI-CPC_0.5_) films exhibited similar behaviors but slightly darker colors than PEI/P­(EI-CPC_0.06_) films. For PEI/P­(EI-CPC_0.8_) and PEI/P­(EI-CPC_1.3_) mixtures, the films were intact when ϕ = 70:30 and 50:50, but the films became brittle at ϕ = 30:70. Upon thermal annealing, most films of PEI/P­(EI-CPC_*x*_) became robust and could be cut into stripes for mechanical testing, except PEI/P­(EI-CPC_1.3_) at ϕ = 30:70.

**Figure 7:**
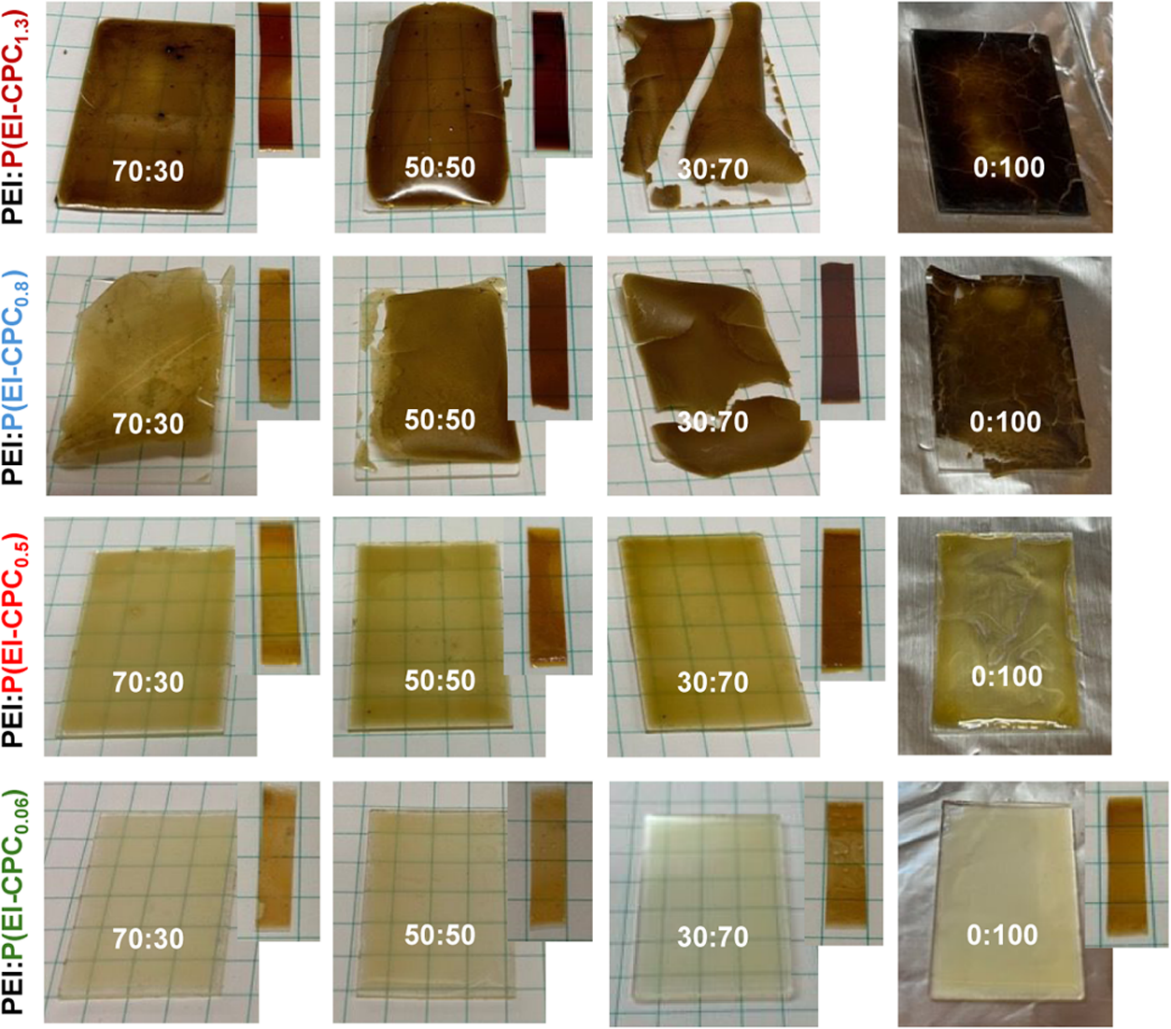
Photographs of solution-cast films of PEI/P­(EI-CPC_*x*_) at different weight mixing ratios. (inset) Films after thermal annealing at 220 °C.

The yellow color of these films could be attributed to the new carbonyls from the 3-CPC moiety, which were conjugated with the aromatics of the PEI backbone. Upon thermal annealing, all films underwent full dehydrochlorination and their color darkened (Figures S9–S11). The brittleness of heavily functionalized P­(EI-CPC_*x*_) films before cross-linking likely stems from the attachment of the 3-CPC moieties, which potentially caused a loss in secondary relaxations.^47–49^ The same effect occurred in films of mixed PEI and P­(EI-CPC_*x*_) at high loadings of the latter.

### Dynamic Mechanical Thermal analysis

The intact stripes were analyzed using dynamic mechanical thermal analysis (DMTA). Virgin PEI displayed an *E*′ of 2.11 ± 0.12 GPa and a distinct *T*_g_ at 226 °C (Figure 8 and Table 2). All films of PEI/P­(EI-CPC_0.06_) retained similar *E*′ values, and their tan δ curves showed almost unnoticeable changes in *T*_g_. PEI/P­(EI-CPC_0.5_) displayed different behaviors at varying mixing ratios. When ϕ = 70:30 and 50:50, the films showed reduced *T*_g_ of 220 and 224 °C, respectively. When ϕ = 30:70, however, PEI/P­(EI-CPC_0.5_) had an increased *T*_g_ of 229 °C, higher than virgin PEI. *E*′ displayed a distinctive rubbery plateau indicative of cross-linking. The modulus decreased initially after *T*_g_ and then increased after 350 °C, with an *E*′ of ∼5.1 MPa at 300 °C (Table 2). Films of PEI/P­(EI-CPC_0.8_) with ϕ = 70:30 exhibited a reduced *T*_g_ of 222 °C. However, films of PEI/P­(EI-CPC_0.8_) with ϕ = 50:50 and 30:70 showed increased *T*_g_ to 235 and 241 °C, with large decreases in tan δ height and a much broader profile. After *T*_g_, the *E*′ curves also displayed distinctive rubbery cross-linking plateaus. The *E*′ values of these PEI/P­(EI-CPC_0.8_) films were ∼6 and ∼16.5 MPa at 300 °C for ϕ = 50:50 and 30:70, respectively. Films of PEI/P­(EI-CPC_1.3_) with ϕ = 30:70 and 50:50 showed increased *T*_g_ of 232 and 237 °C, respectively, and tan δ displayed similar characteristics of broadening and reduced height. The *E*′ values of these films at 100 °C were ∼2.4 GPa (Table 2), slightly higher than those of virgin PEI. PEI/P­(EI-CPC_1.3_) also displayed induced rubbery plateaus in *E*′ curves, giving *E* **′** of ∼5.5 and ∼20 MPa at 300 °C at ϕ = 30:70 and 50:50, respectively. All films with plateaus in the DMTA test showed signs of oxidation at the end of the test (Figure S12).

**Figure 8:**
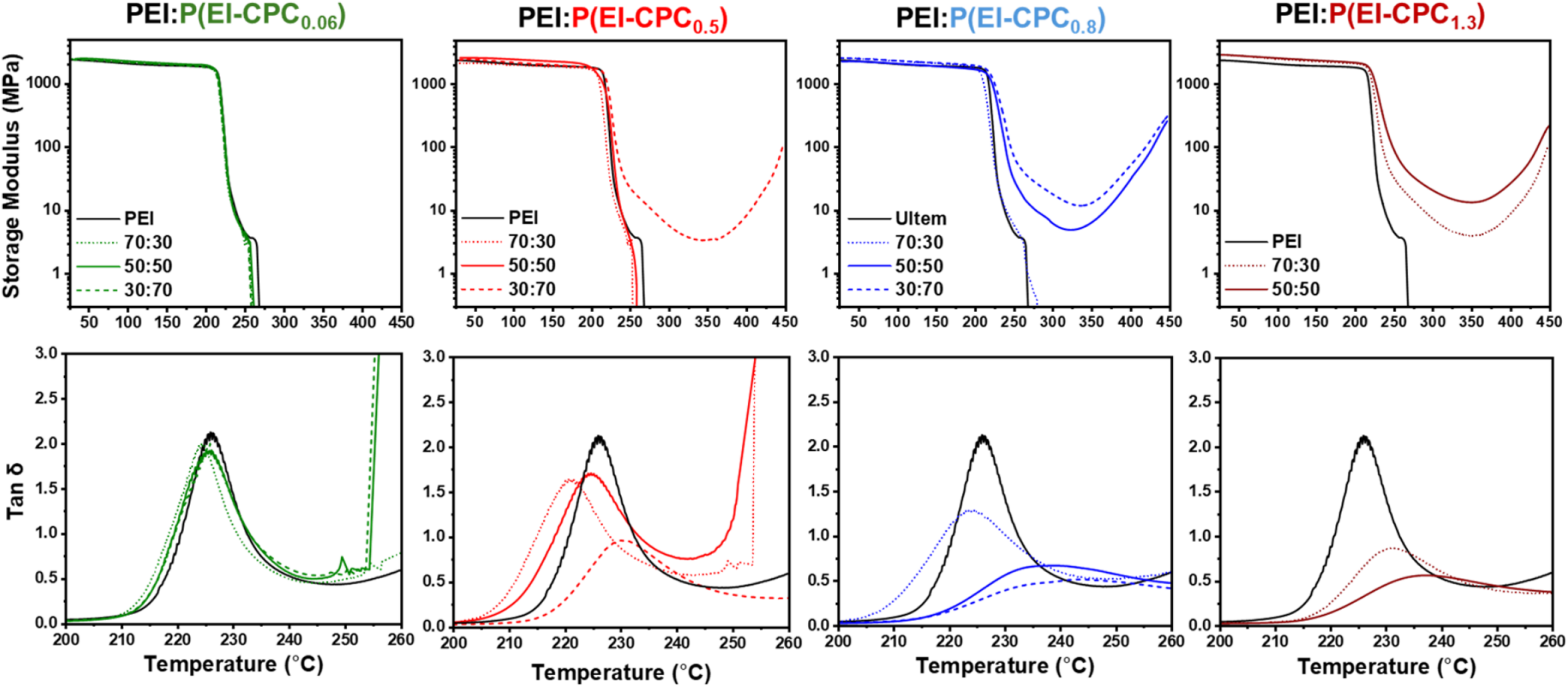
DMTA of annealed PEI/P­(EI-CPC_*x*_) films at different weight mixing fractions.

**Table 2:** Physical Properties of PEI/P­(EI-CPC_*x*_) Compared to other Polymers

		*T*_g_ (°C)					
	mixing ratio*	DSC	DMA	*T*_d_ (°C)	*E*′ (100 °C) (GPa)	*E*′ (300 °C) (MPa)	cross-link density (mol/m^3^)
PEI/P(EI-CPC_1.3_)	30:70			530			
	50:50	222	237	539	2.43 ± 0.12	20.2 ± 0.78	1560 ± 56
	70:30	220	232	546	2.32 ± 0.10	5.46 ± 0.52	421 ± 40
PEI/P(EI-CPC_0.8_)	30:70		241	533	2.30 ± 0.09	16.5 ± 1.2	1270 ± 93
	50:50	221	235	540	2.23 ± 0.19	6.01 ± 0.40	463 ± 31
	70:30	210	222	545	2.15 ± 0.15		
PEI/P(EI-CPC_0.5_)	30:70	220	230	542	2.22 ± 0.12	5.13 ± 0.48	395 ± 34
	50:50	214	224	547	2.19 ± 0.08		
	70:30	212	220	552	2.24 ± 0.14		
PEI/P(EI-CPC_0.06_)	0:100	218	225	539	2.15 ± 0.16		
	30:70	217	226	545	2.13 ± 0.11		
	50:50	216	225	548	2.18 ± 0.08		
	70:30	215	224	551	2.16 ± 0.13		
PEI	n/a	217	226	554	2.11 ± 0.12		
Kapton	n/a	∼385	370	574	2.30 ± 0.20	1550	
X-PEI-8*	n/a	224	238	544	1.76	2.29	177

We used *E*′ values at 300 °C to determine the degree of cross-linking. We selected this temperature because the onset of *T*_d_ occurred at temperatures ≥300 °C for all films (Figure S13), and above this temperature, the films might be subject to too much oxidation and carbonization.^14^ The *E*′ values were in the range of ∼5.1–20 MPa for all films, and the cross-linking density was determined using eq 1. PEI/P­(EI-CPC_1.3_) films at ϕ = 50:50 showed the highest cross-linking density of 1560 mol/m^3^, an order of magnitude greater than our previous report for azide functionalized PEI (X-PEI-8) (Table 2).^14^ This cross-linking density followed a somewhat linear correlation with the number of 3-CPC moieties (Figure S14).

When P­(EI-CPC_*x*_) was mixed with virgin PEI, most films exhibited similar *E*′ of ∼2.1 GPa at 100 °C, and the values were slightly higher at higher cross-linking densities. Based on tan δ, *T*_g_ was directly affected by the number of 3-CPC moieties in the PEI/P­(EI-CPC_*x*_) films. The moieties appeared to negatively affect the number of interchain interactions of the PEI chain when attached, decreasing the overall *T*_g_. This effect was demonstrated by Ferreira et al. when attaching different acyl halides of varying carbon numbers, showing a dependence of *T*_g_ on the carbon number of acyl halide.^27^ However, upon heating, the 3-CPC moieties were dehydrochlorinated and cross-linked within our films, which increased *T*_g_. When the number of 3-CPC moieties was low, this cross-linking effect was insufficient to counteract the decreased entanglement, which decreased overall *T*_g_. When the number of attached 3-CPC moieties was high, the cross-linking effect became sufficient to overcome the loss of interchain interaction, increasing *T*_g_. When P­(EI-CPC_*x*_) was mixed with virgin PEI, the films were cross-linked, showing broader and less intense Tan δ peaks. The broad tan δ indicates the inhomogeneity of *T*_g_ and a wide temperature range of transitions caused by nonuniform cross-linking.^50–52^

### Thermal Analysis

DSC was used to determine the *T*_g_ of PEI/P­(EI-CPC_*x*_) films. Virgin PEI showed a *T*_g_ value of 217 °C (Figure 9 and Table 2), similar to previous reports.^27,28,14^ PEI/P­(EI-CPC_0.06_) of all mixing ratios showed insignificant changes in *T*_g_. In comparison, PEI/P­(EI-CPC_0.5_) showed slightly reduced *T*_g_ of 212 and 214 °C at ϕ = 70:30 and 50:50, respectively, but at ϕ = 30:70, the *T*_g_ increased to 220 °C. Compared to virgin PEI, *T*_g_ of PEI/P­(EI-CPC_0.8_) exhibited a major decrease to 210 °C at ϕ = 70:30 but a noticeably higher value of 221 °C at ϕ = 50:50. PEI/P­(EI-CPC_1.3_) of varying mixing ratios displayed increased *T*_g_, ranging from 220 to 222 °C. Below a certain threshold of 3-CPC functionalization (<0.35), the attached 3-CPC moieties likely negatively affected the polymer chain entanglement and packing, thus reducing the *T*_g_ values of the polymers (Figure S15). Above this threshold (>0.35), the number of 3-CPC moieties allowed for sufficient polymer cross-linking, counteracting the loss of interchain interaction caused by the side chains, increasing the *T*_g_. In addition, as evidenced by DSC (Figure 9), both PEI/P­(EI-CPC_0.8_) and PEI/P­(EI-CPC_1.3_) at ϕ = 30:70 exhibited a broader thermal event, suggesting that some of the cross-linking linkages were degraded. This effect was further supported by the second heating of the same material, which demonstrated lower *T*_g_ (Figure S16).

**Figure 9:**
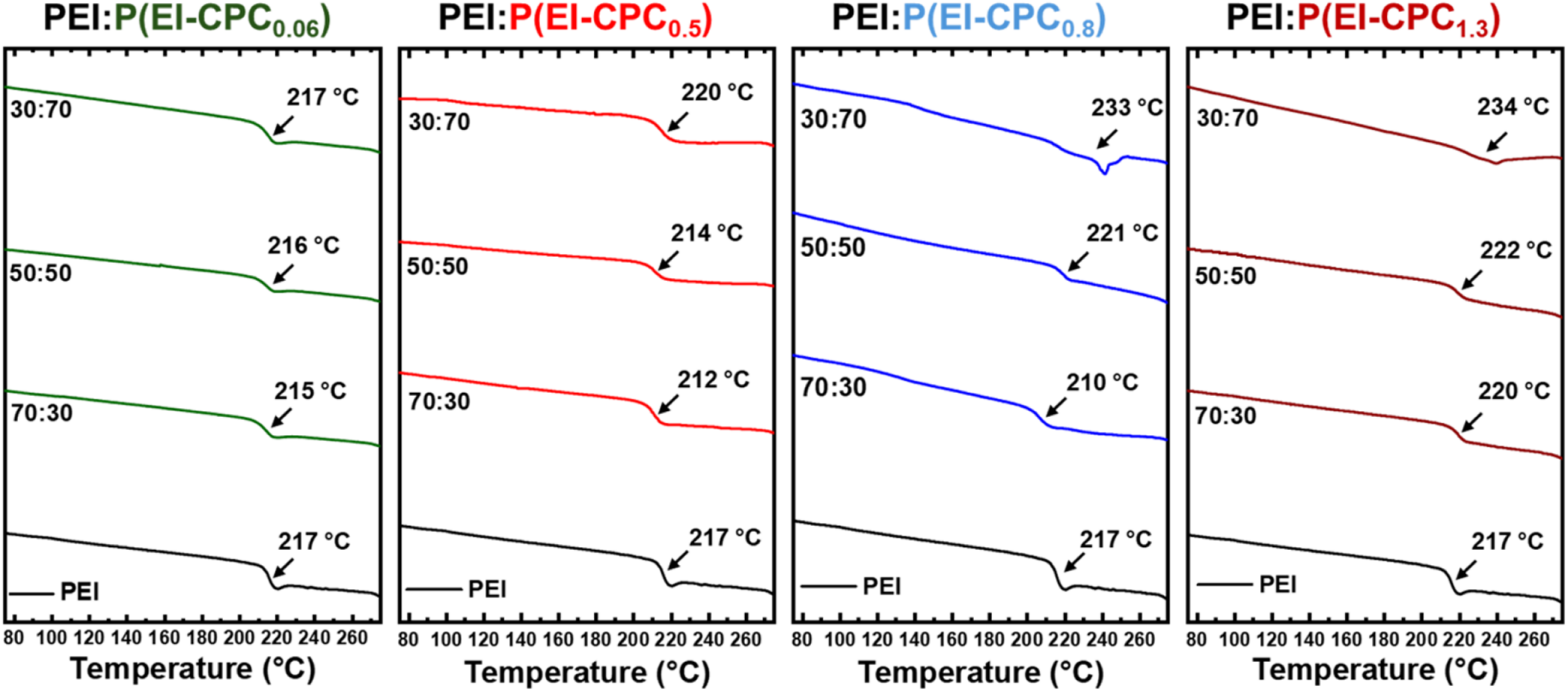
DSC of all PEI/P­(EI-CPC_*x*_) solution cast films after annealing at 220 °C.

### Solvent Resistance

To understand the effect of cross-linking on solvent resistance, all PEI/P­(EI-CPC_*x*_) films were immersed in five solvents, including NMP, DMF, CHCl_3_, DCM, and THF, for 7 days. Virgin PEI was completely soluble in all these solvents after 7 days (Figure 10). As a benchmark, a solvent-resistant cross-linked film of X-PEI-8 from our previous report^14^ underwent the same solvent testing. After 7 days, it was not dissolved but swollen (∼6–55%) by these solvents. THF caused the least swelling, while CHCl_3_ caused the most (Table 3). For PEI/P­(EI-CPC_*x*_) without thermal annealing, all films were soluble in these solvents. After thermal annealing at 220 °C, the films showed different levels of solvent resistance. Interestingly, for all PEI/P­(EI-CPC_*x*_) films that showed no signs of cross-linking from the DMTA tests (e.g., PEI/P­(EI-CPC_0.05_) 50:50), the solvent resistance was poor, and the films disintegrated in the solvents (Figure S17). Among the films with an induced rubber plateau, PEI/P­(EI-CPC_0.5_) with ϕ = 30:70 showed less swelling than X-PEI-8, and similarly, THF caused the least amount of swelling while CHCl_3_ the most. PEI/P­(EI-CPC_0.8_) with ϕ = 50:50 and PEI/P­(EI-CPC_1.3_) with ϕ = 70:30 showed similar solvent resistance, and the swelling ratio was in the range of ∼1.5–28% depending on the solvent. In contrast, PEI/P­(EI-CPC_0.8_) with ϕ = 30:70 and PEI/P­(EI-CPC_1.3_) with ϕ = 50:50 displayed the highest solvent resistance, and the swelling ratio was as low as ∼0.1% in most solvents, except for in CHCl_3_ and DCM with swelling ratios up to ∼9.3%. Notably, these two films showed better solvent resistance to DMF and NMP than Kapton did, and the latter was swollen by ∼4% in DMF and NMP.^53^

**Figure 10:**
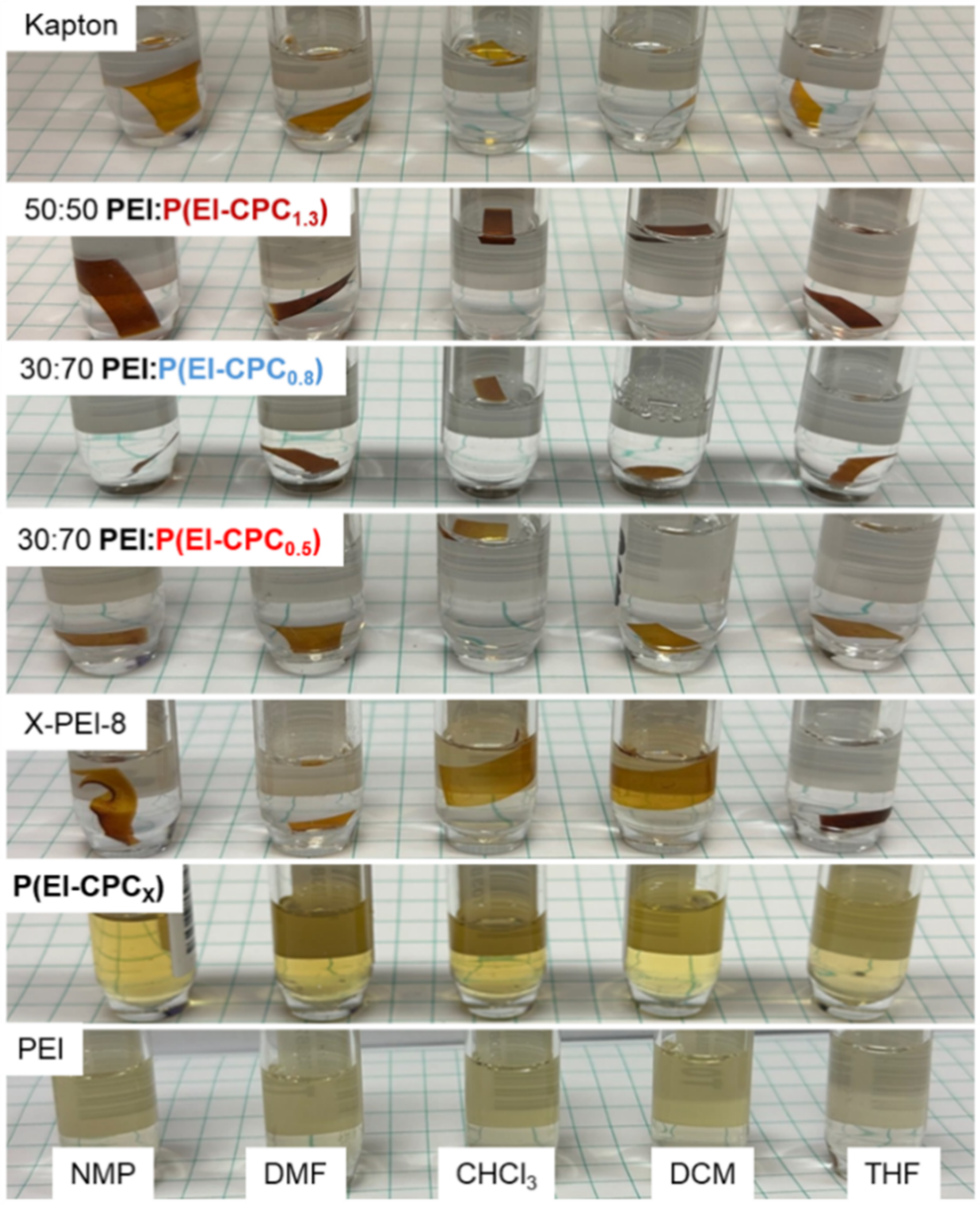
Solvent swelling test by submerging the polymer films in different solvents for 7 days.

**Table 3:** Swelling Properties of PEI/P­(EI-CPC_*x*_) Compared to other Polymers

		swelling ratio (%)				
	mixing ratio*	NMP	DMF	CHCl_3_	DCM	THF
PEI/P(EI-CPC_1.3_)	50:50	∼ 0.1	∼ 0.1	2.3	∼ 0.5	∼ 0.1
	70:30	13.6	7.5	22.3	11.6	1.4
PEI/P(EI-CPC_0.8_)	30:70	∼ 1	2.3	9.3	6.8	∼ 0.4
	50:50	11.1	6.6	28.1	19.7	2.2
PEI/P(EI-CPC_0.5_)	30:70	12.2	6.4	19.1	15.9	2.6
Kapton	n/a	4	3.7	∼ 0.3	∼ 0.3	∼ 0.4
X-PEI-8*	n/a	45.4	14.6	54.8	46.6	6.4

The different levels of solvent resistance of thermally annealed PEI/P­(EI-CPC_*x*_) films depended on the cross-linking density. As the cross-linking density of the films decreased, their swelling ratio increased. When the cross-linking density was >1200 mol/m^3^, the swelling ratios of the films were <10% (e.g., PEI/P­(EI-CPC_1.3_) with ϕ = 50:50 and PEI/P­(EI-CPC_0.8_) with ϕ = 30:70). Some of the films showed extremely low swelling ratios of <1%. Films with a cross-linking density of 395–460 mol/m^3^, the swelling ratios were much higher (up to ∼28%). This behavior was witnessed with X-PEI-8 in a previous report, in which a decreasing cross-linking density reduced the solvent resistance.^14^ When X-PEI-8 underwent the same treatment as PEI/P­(EI-CPC_*x*_), swelling ratios were an order of magnitude greater, likely due to the much lower cross-linking density of X-PEI-8 (∼177 mol/m^3^) than that of PEI/P­(EI-CPC_*x*_) (ranging from 395 to 1560 mol/m^3^).

Although PEI/P­(EI-CPC_*x*_) films showed great solvent resistance, all films exhibited mass loss during the test (Table S2). With cross-linking densities >1200 mol/m^3^, PEI/P­(EI-CPC_1.3_) 50:50 and PEI/P­(EI-CPC_0.8_) 30:70 showed negligible mass loss in NMP, DMF, and THF but much more mass loss (∼10.3–40.1 wt %) in DCM and CHCl_3_ after 7 days. Noticeably, films with lower cross-linking densities showed much greater mass loss in all solvents. As suggested by the broad tan δ curves (Figure 8), these films likely were cross-linked nonuniformly, and the less cross-linked chains leached out of the films during the solvent resistance test, embrittling the films. We submerged all films in DCM and measured their residual mass at various time points (Figure S18). For PEI/P­(EI-CPC_1.3_) 50:50 and PEI/P­(EI-CPC_0.8_) 30:70 with a cross-linking density of >1200 mol/m^3^, the mass loss was minor (<5 wt %) after ∼6 h and ∼38–40 wt % after 7 days (Figure S18). The rest films with a cross-linking density in the range of 395–460 mol/m^3^ lost ∼35–45 wt % mass after 2 h and plateaued at ∼48–50 wt % after 7 days (Figure S18).

Comparing PEI/P­(EI-CPC_*x*_) with the highest cross-linking density to Kapton films, the latter swelled the most in NMP (∼4%), whereas the former swelled the most in CHCl_3_ and DCM (∼2.3%). DCM and CHCl_3_ are known to have the best affinities (or lowest χ values) to PEI, as shown in our previous work,^14^ thus causing the most swelling and mass loss for cross-linked PEI/P­(EI-CPC_*x*_). Therefore, cross-linked PEI/P­(EI-CPC_*x*_) exhibited excellent swelling resistance comparable to Kapton and an order of magnitude better than X-PEI-8, at the highest cross-linking density.

## Conclusions

In conclusion, Freidel–Crafts acylation of PEI with 3-CPC resulted in a functional polymer of P­(EI-CPC_*x*_). Thermal annealing induced dehydrochlorination of the 3-CPC moieties in P­(EI-CPC_*x*_) and produced a densely cross-linked network with enhanced solvent resistance. However, P­(EI-CPC_*x*_) showed reduced mechanical strength and was susceptible to cracking. After mixing with virgin PEI, P­(EI-CPC_*x*_) and PEI cross-linked to produce mechanically strong films, showing high solvent resistance to NMP, DMF, CHCl_3_, DCM, and THF. Among all the test systems, PEI/P­(EI-CPC_0.8_) with ϕ = 30:70 and PEI/P­(EI-CPC_1.3_) with ϕ = 50:50 displayed the highest cross-linking densities, thus the highest *T*_g_, storage moduli, and solvent resistance. The cross-linking density increased with the concentration of CPC moieties.

This work reports an effective approach to functionalize PEI into P­(EI-CPC_*x*_) at the backbone using reactive 3-CPC moieties. The functionalized P­(EI-CPC_*x*_) could effectively modulate thermal, mechanical, and solvent-resistant properties via cross-linking. This approach highlights a new avenue for postpolymerization functionalization of engineering plastics. Owing to the simplicity of the Friedel–Crafts reaction, the method can be extended to synthesizing block copolymers, bottle brushes, and vitrimers from engineering plastics. With extreme solvent and heat resistance, the new polymers could find use in biomedicals,^54,55^ electronics,^56–60^ airspace and aerospace,^61,62^ and filtration/separatory applications,^63,64^ where extreme thermomechanical properties and solvent resistance are required.

## Supplementary Material



## Notes

Additional experimental details, methods, photographs of polymer films and solvent testing, ^1^H NMR spectra, reaction schemes, scanning electron micrographs, and DSC/TGA traces (PDF)
