# Supplementary material for: Hyper Cross-linked Poly(ether imide) via Friedel–Crafts Acylation and Dehydrochlorination
Source: Macromolecules. 2025 Jun 25;58(13):6440–51. doi: 10.1021/acs.macromol.5c00532 (PMC12257598; doi:10.1021/acs.macromol.5c00532)
Supplement: Supplementary file 1 [file ma5c00532_si_001.pdf]

Supporting Information for:

Hyper Crosslinked Polyetherimide via Friedel—Crafts Acylation and Dehydrochlorination

Connor S. Thompson,[1] *Carlos Posada,*[1] *and Guoliang Liu*[1,2,3,4*]

[1]Department of Chemistry, Virginia Tech, Blacksburg, VA 24061, USA

[2]Macromolecules Innovation Institute, Virginia Tech, Blacksburg, VA 24061, USA

[3]Division of Nanoscience, Academy of Integrated Science, Virginia Tech, Blacksburg, VA 24061, USA

[4]Department of Chemical Engineering, Department of Materials Science and Engineering, Virginia Tech, Blacksburg, VA 24061, USA

*Corresponding authors, E-mail: gliu1@vt.edu

PEI and PEI:P(EI-$CPC_{1.3}$) 50:50 (1 wt. % in $CHCl_3$) were solution-cast and dried into films on glass slides. The films were characterized using phase contrast optical microscopy (Nikon Eclipse LV100), showing a single polymer phase without any haziness. Additionally, small-angle light scattering revealed no scattering patterns, confirming the uniform mixing of PEI and P(EI-$CPC_{1.3}$) across the films. Thus, we concluded that PEI and P(EI-CPC_x) were fully miscible.

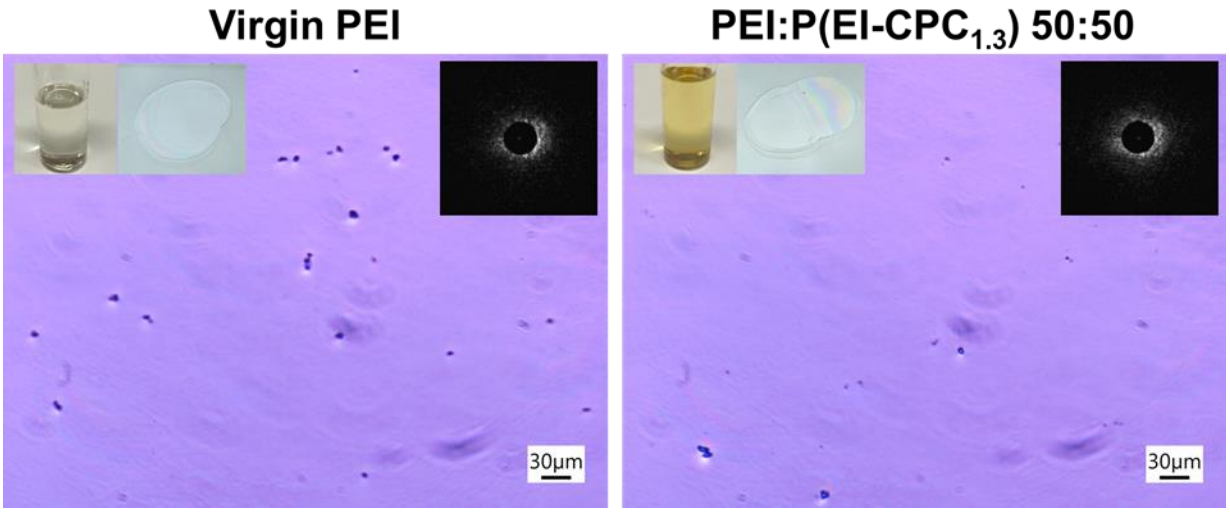

Figure S1. Phase contrast optical microscopy of (left) PEI and (right) PEI:P(EI-$CPC_{1.3}$) 50:50. (left insets) Photographs of solutions and thin films on glass slides. (right insets) Small angle laser light scattering patterns.

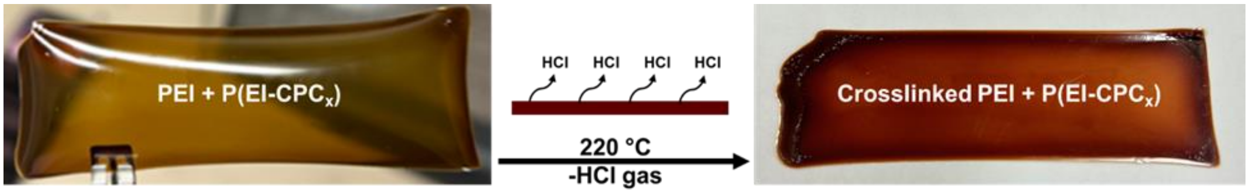

Figure S2. Thermal annealing of PEI:P(EI-CPC_x) crosslinked the polymers and released HCl gas.

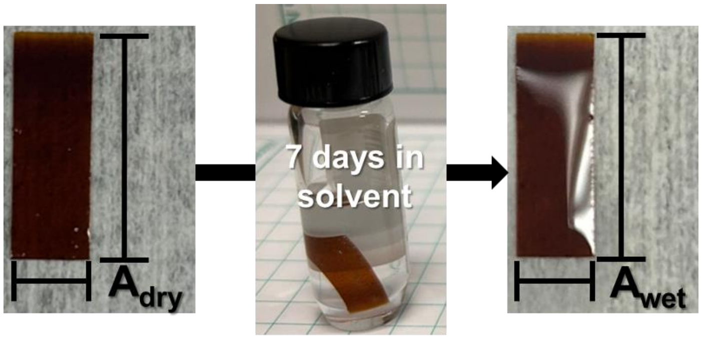

Figure S3. Thin films of PEI:P(EI-CPC$_x$) before and after exposure to solvent for 7 days.

To determine the degree of functionalization, P(EI-CPC$_x$) was analyzed via ^{1}H NMR using an internal standard of dichloromethane (DCM). DCM was chosen because its proton peak position (5.31 ppm) is far away from any known peaks in P(EI-CPC$_x$). The protons next to the C-Cl bonds in the 3-CPC moieties were located at 3.95 ppm. Direct integration of these protons enabled us to calculate the molar ratio between the 3-CPC moieties and the added amount of DCM. The degree of functionalization was calculated as the amount of 3-CPC moieties with respect to the amount of PEI monomers.

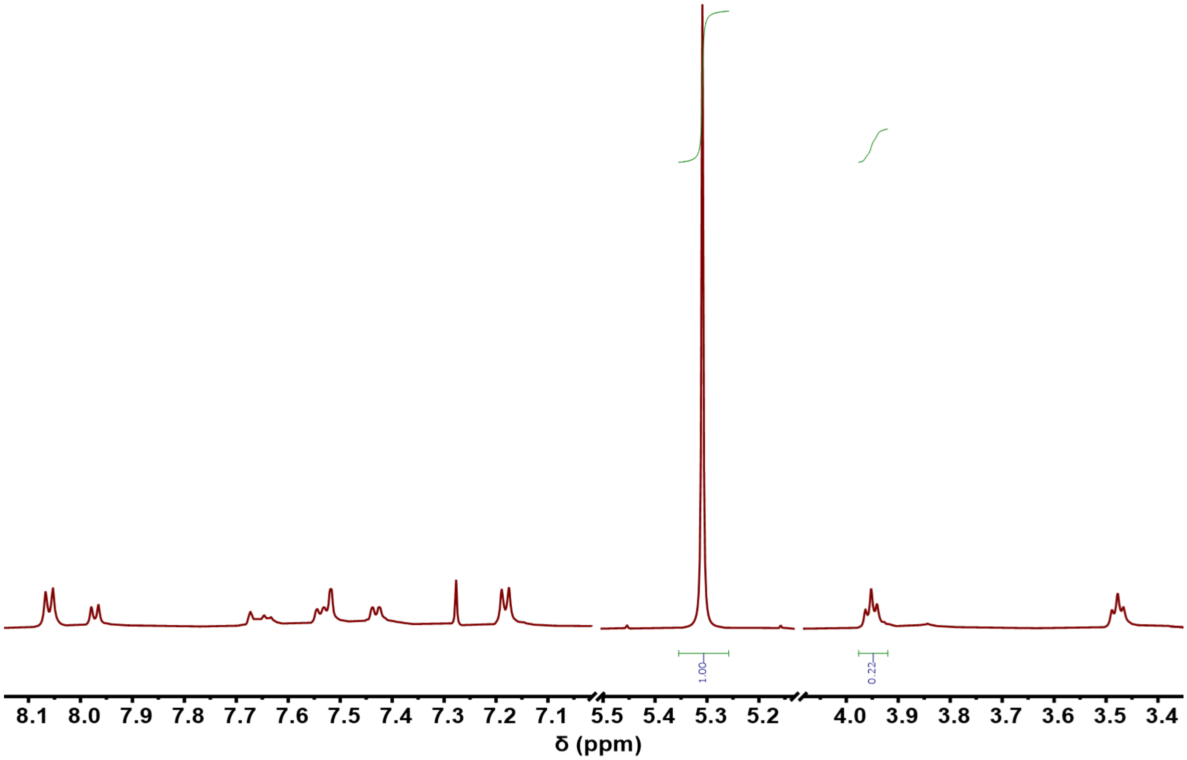

Figure S4. ^{1}H NMR of P(EI-CPC$_{1.3}$) (27.51 mg) with an internal standard of DCM (14 μL). The mass of the polymer and the volume of DCM were fixed to determine the proton intensity ratios.

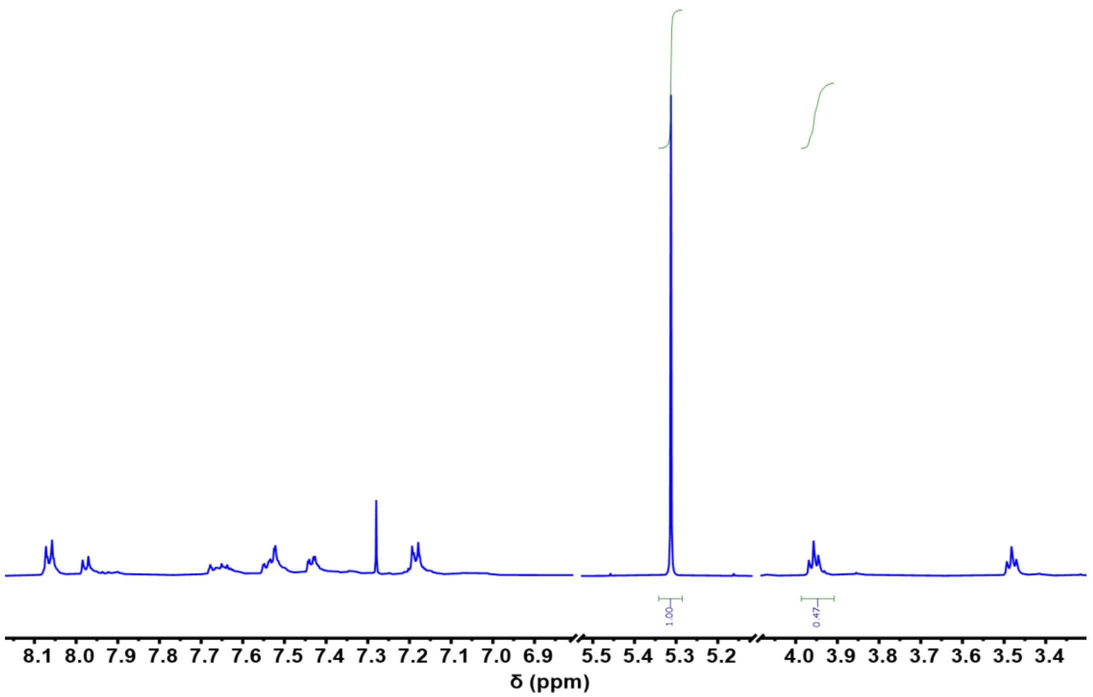

Figure S5. ^{1}H NMR of P(EI-CPC$_{0.8}$) (24.15 mg) with a DCM standard (4 μL).

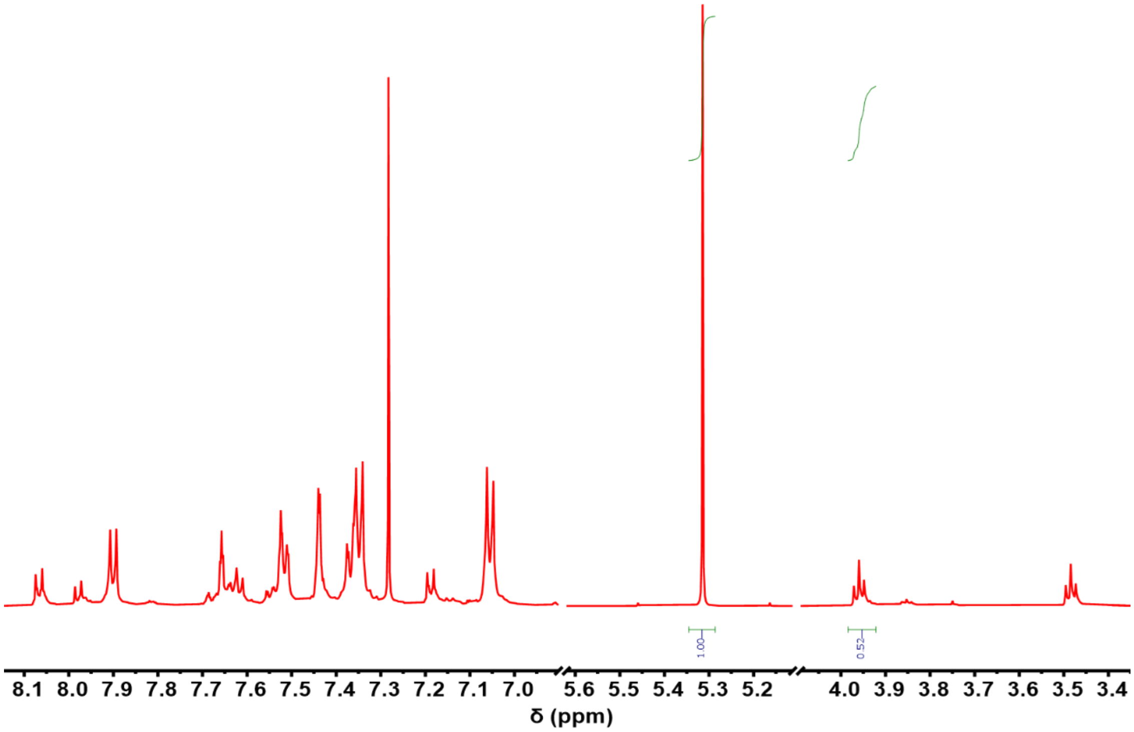

Figure S6. ^{1}H NMR of P(EI-CPC$_{0.6}$) (39.39 mg) with a DCM standard (4 μL).

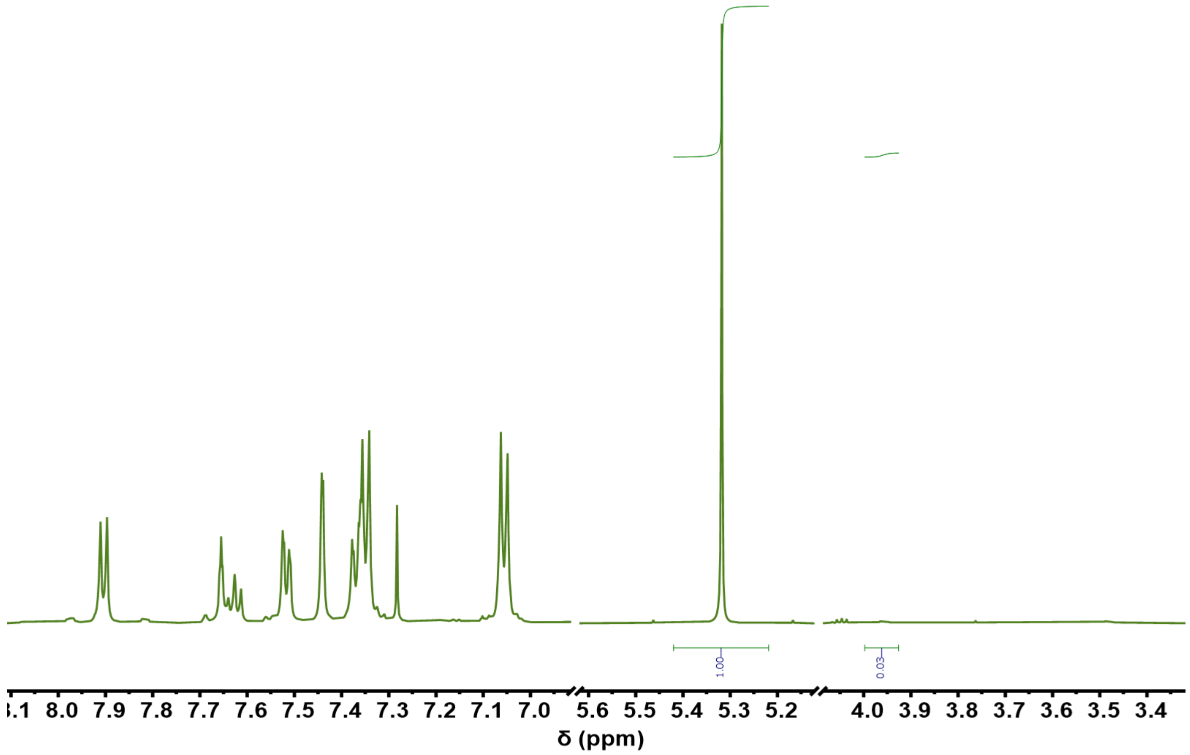

Figure S7. ^{1}H NMR of P(EI-CPC$_{0.06}$) (19.91 mg) with a DCM standard (4 μL).

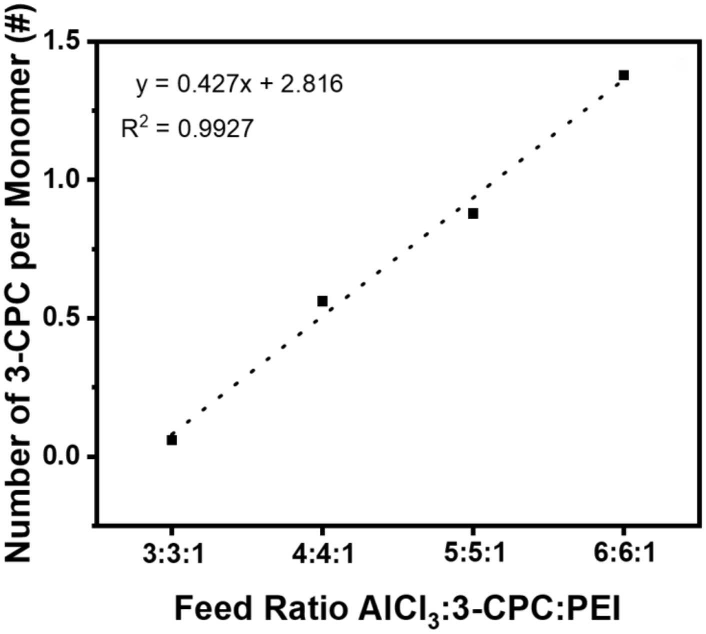

Figure S8. The number of attached 3-CPC moieties increased linearly with the molar ratio of $AlCl_3$: 3-CPC:PEI used in the functionalization reaction.

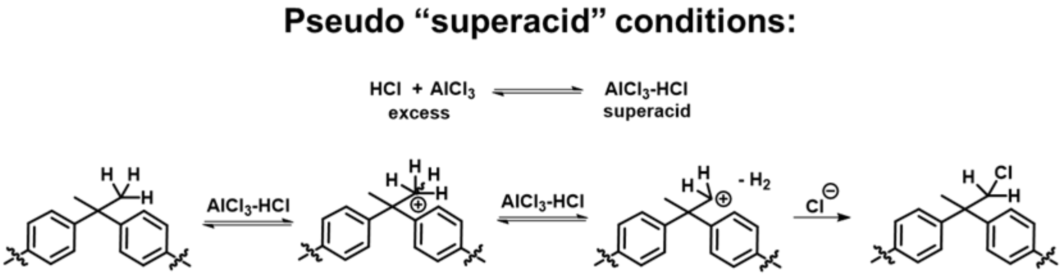

Scheme S1. Proposed mechanism of PEI methyl group halogenation under “superacid” conditions.

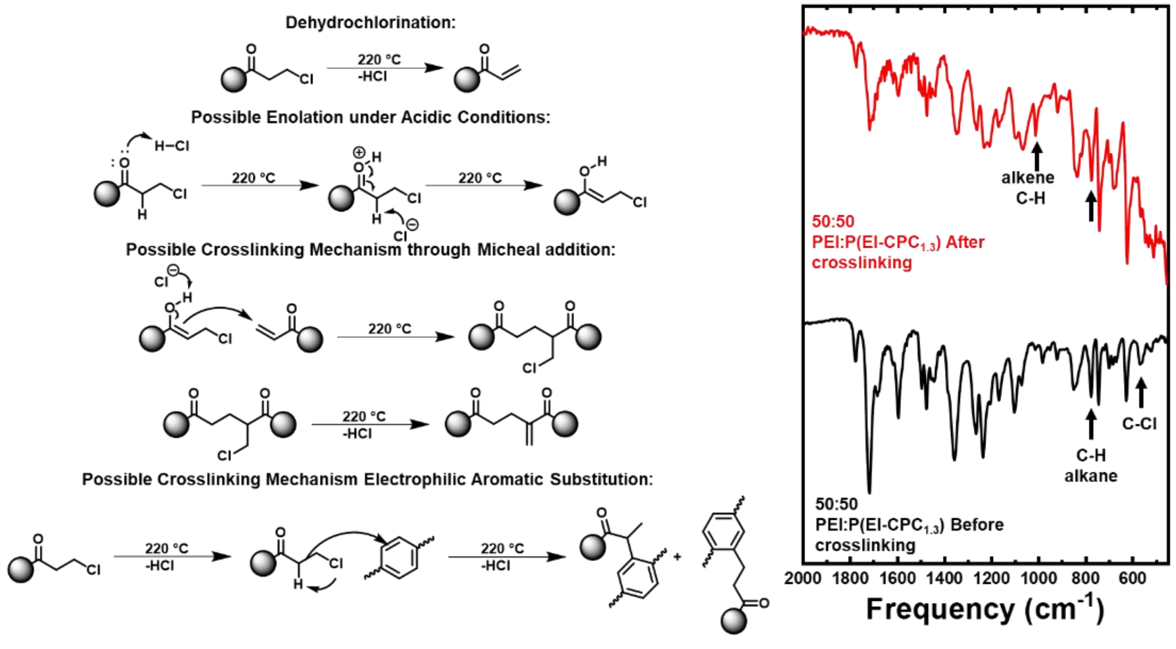

Scheme S2. The proposed crosslinking mechanisms via possible reactive intermediates that occur during dehydrochlorination, such as enolation, Michael addition, and electrophilic aromatic substitution (EAS). The FTIR spectra show new peaks associated with C-H of alkenes and the reduction of C-H alkanes.

To determine the conversion ratio of the dehydrochlorination reaction, Energy Dispersive Spectroscopy (EDS) was performed on three Films (PEI:P(EI-$CPC_{1.3}$) 70:30, PEI:P(EI-$CPC_{0.8}$) 50:50, and PEI:P(EI-$CPC_{0.5}$) 30:70), before and after annealing, using an SEM (JEOL IT-500HR). Each test was conducted using a working distance of 10 mm at 10 kV. Films were sputtered with 4 nm of palladium and platinum to increase their conductivity. The EDS analyses provided the amount of chlorine in the annealed and nonannealed films.

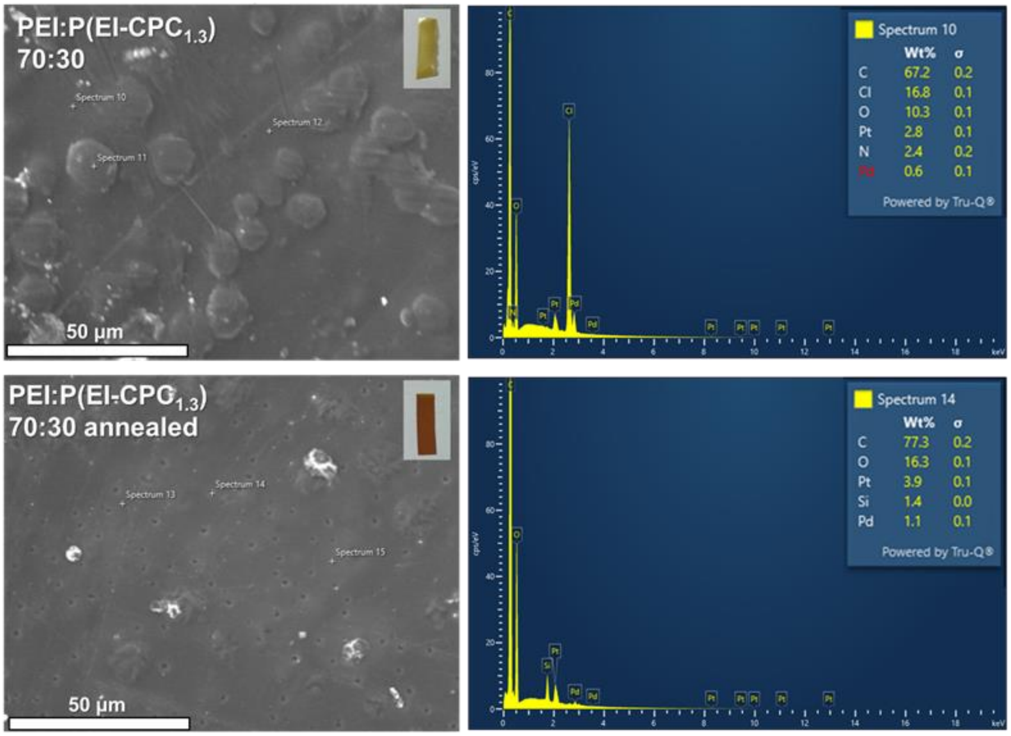

Figure S9. EDS of unannealed (~17 wt. % Cl, top panels) and annealed (0 wt. % Cl, bottom panels) PEI:P(EI-$CPC_{1.3}$) 70:30 films, demonstrating full dehydrochlorination.

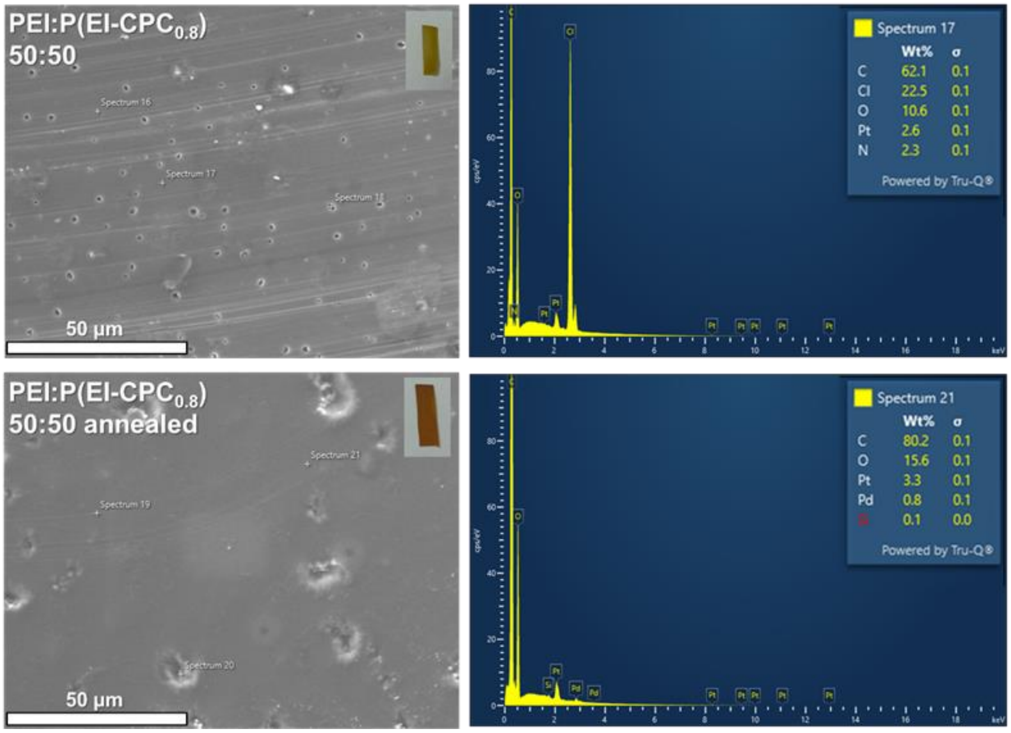

Figure S10. EDS of unannealed (~23 wt. % Cl, top panels) and annealed (0 wt. % Cl, bottom) films of PEI:P(EI-CPC$_{0.8}$) 50:50, demonstrating full dehydrochlorination.

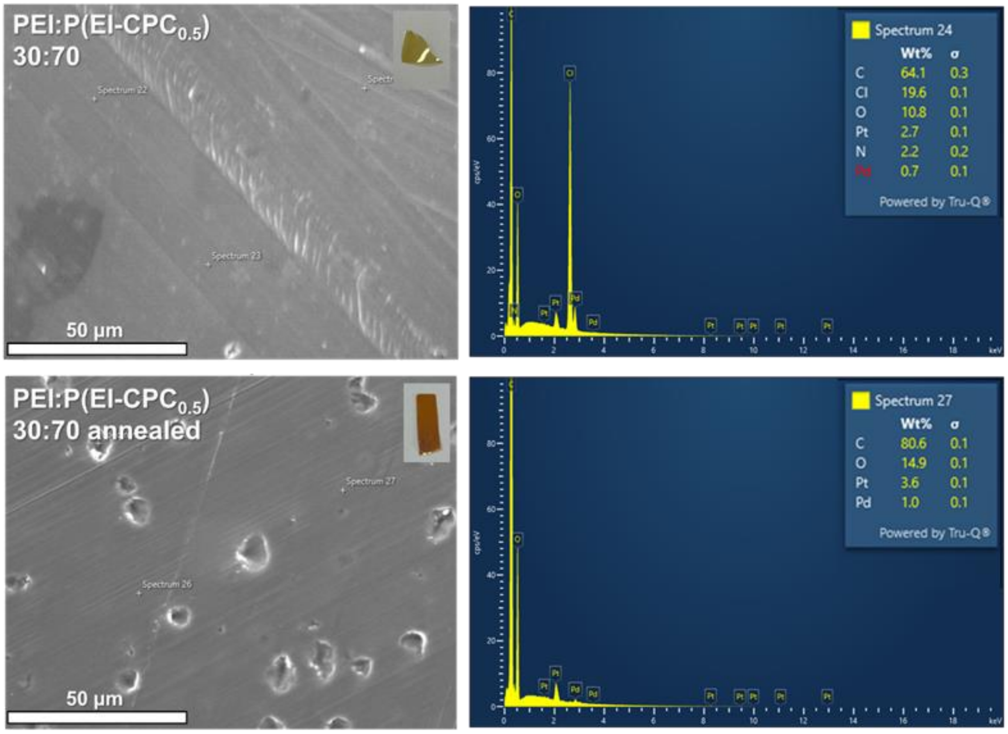

Figure S11. EDS of unannealed (~20 wt. % Cl, top panels) and annealed (0 wt. % Cl, bottom) films of PEI:P(EI-CPC$_{0.5}$) 30:70, demonstrating full dehydrochlorination.

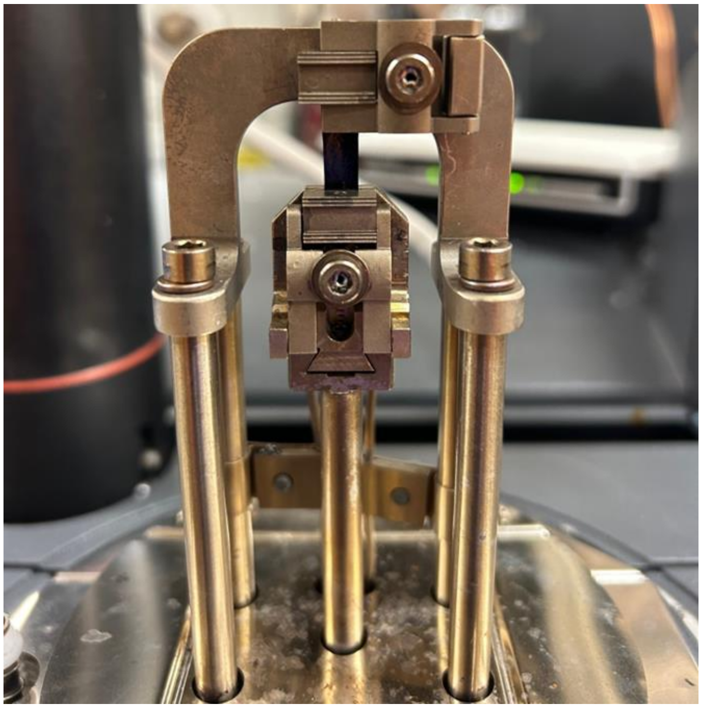

Figure S12. A crosslinked film of PEI:P(EI-CPC$_x$) after DMTA test showed partial oxidation upon reaching 450 °C.

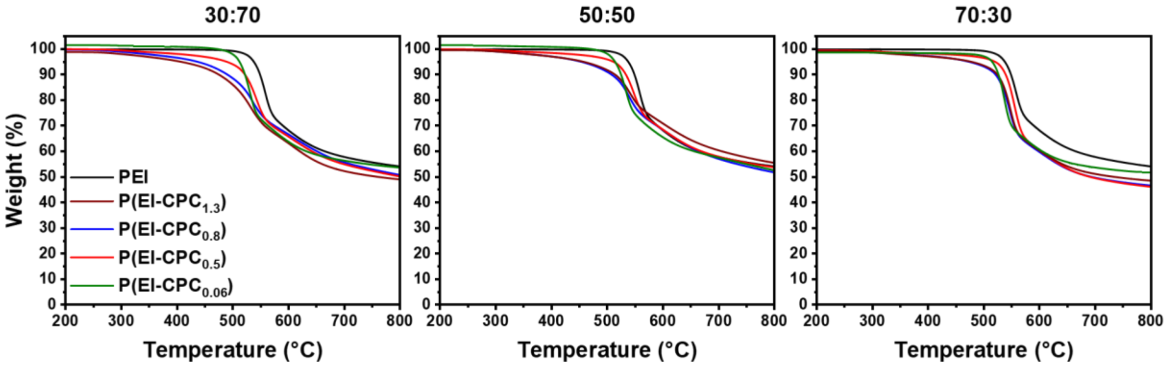

Figure S13. Thermogravimetric analysis of crosslinked PEI:P(EI-CPC$_x$) films after annealing at 220 °C for 6 hours.

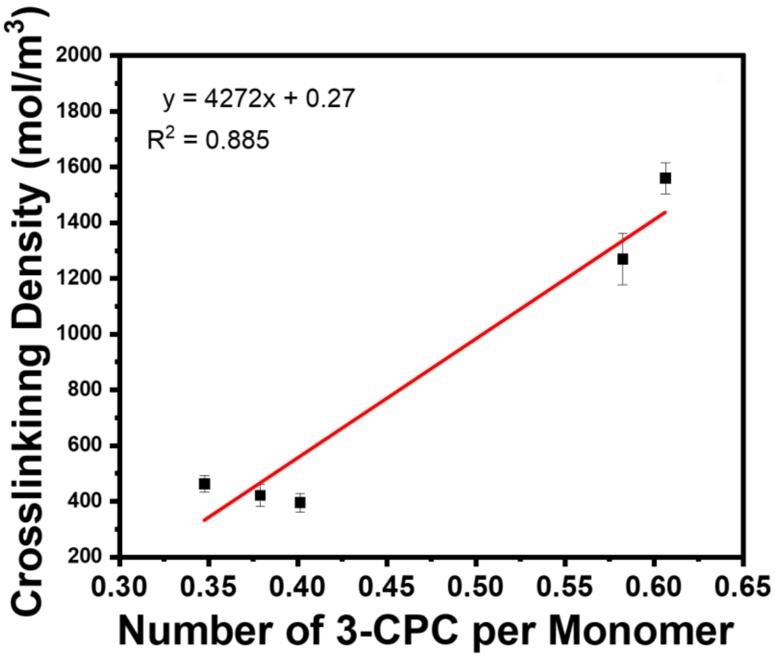

Figure S14. Crosslinking density as a function of the number of 3-CPC moieties per monomer in functionalized P(EI-CPC$_x$).

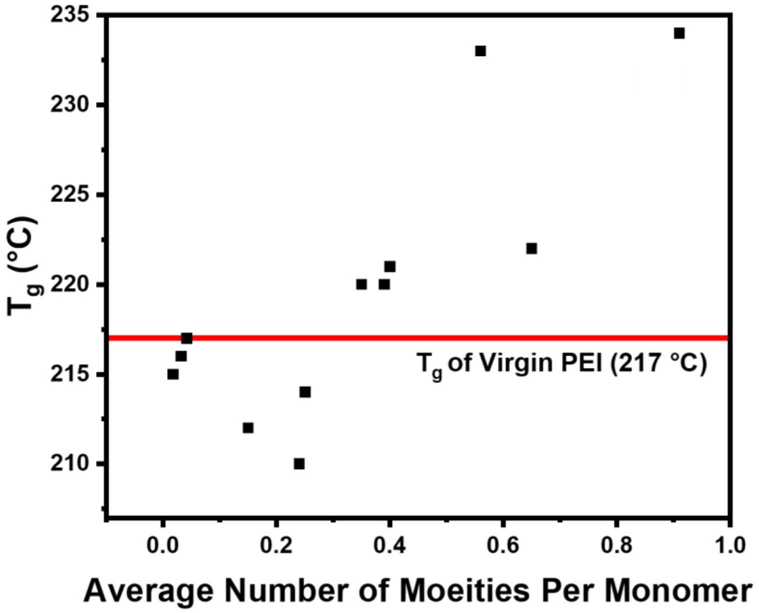

Figure S15. Glass transition temperature of PEI:P(EI-CPC$_x$) films, as determined from tan δ analysis, with respect to the average number of 3-CPC moieties per monomer in functionalized P(EI-CPC$_x$).

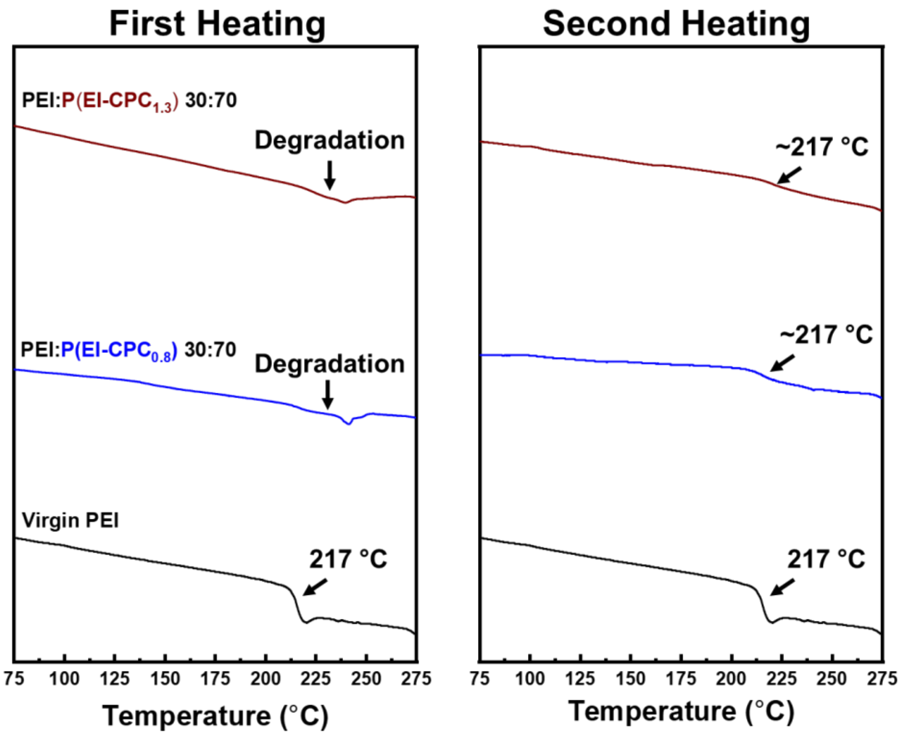

Figure S16. DSC of 30:70 mixing of PEI:P(EI-CPC$_{1.3}$), PEI:P(EI-CPC$_{0.8}$), and virgin PEI films. The first heating ramp showed a thermal event in the range of 220-250 °C, while a second heating ramp showed a glass transition at ~ 217 °C, similar to virgin PEI.

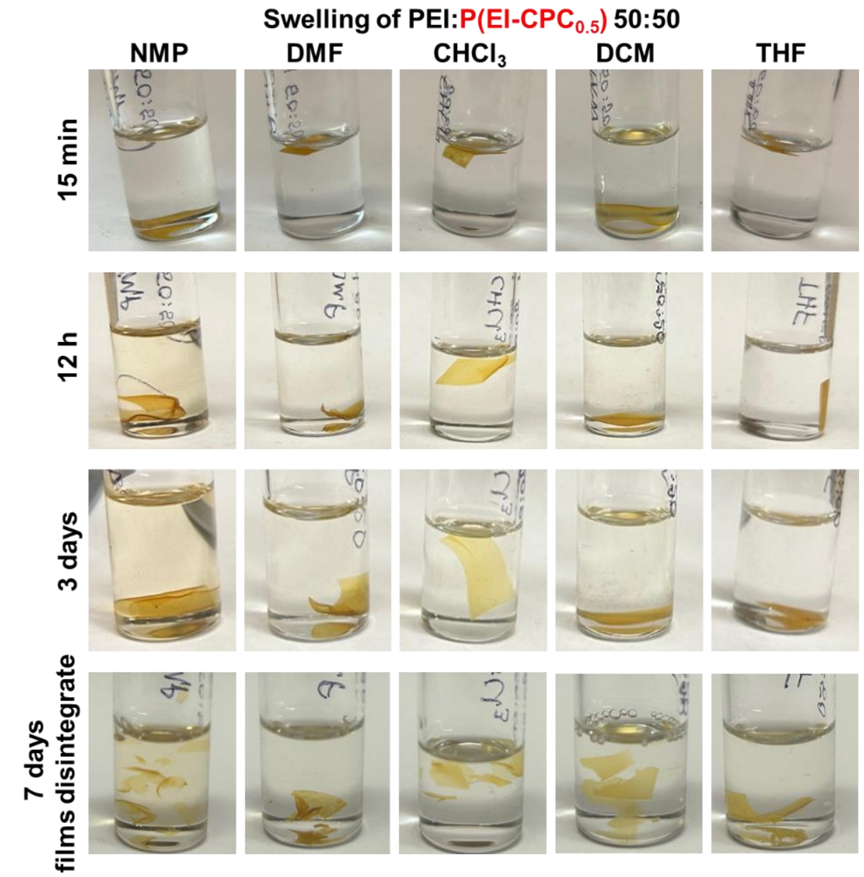

Figure S17. Films of PEI:P(EI-CPC$_{0.5}$) 50:50 were swollen when submerged in solvent and disintegrated after a few days, showing weak solvent resistance.

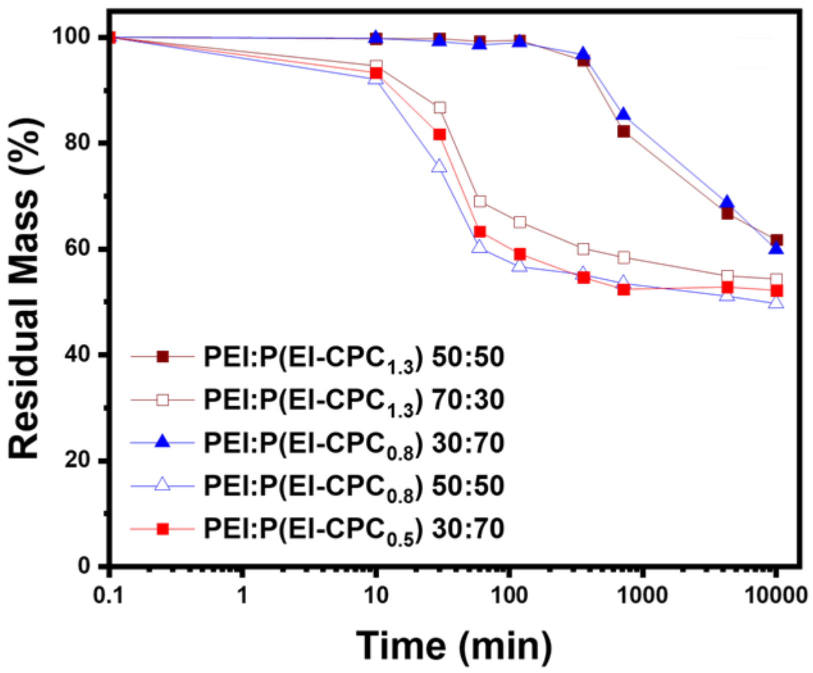

Figure S18. Residual mass of crosslinked PEI:P(EI-CPC$_x$) films after submerging in DCM for various lengths of time.

Table S1. ^{1}H NMR shift of PEI before and after the Friedel-Crafts acylation using 3-CPC.

Moiety	Peak No.	δ (ppm)	
		Before	After
Imide	1	7.90	8.06
	2	7.37	7.54
	3	7.44	7.52
BPA	4	7.05	7.18
	5	7.35	7.43
Methyl	6	1.77	2.45
Phenyl	7	7.51	7.92
	8	7.61	7.64
	9	7.66	7.67

Table S2: Mass loss of PEI:P(EI-CPC_x) films during the 7-day solvent resistance testing.

	Mixing ratio*	Mass Loss (wt. %)				
		NMP	DMF	$CHCl_3$	DCM	THF
PEI:P(EI-$CPC_{1.3}$)	50:50	1.76	3.39	13.9	38.3	2.66
	70:30	9.6	11.4	40.2	45.7	3.07
PEI:P(EI-$CPC_{0.8}$)	30:70	2.50	1.59	10.3	40.1	1.95
	50:50	24.0	16.3	45.9	50.4	7.53
PEI:P(EI-$CPC_{0.5}$)	30:70	17.9	14.4	38.1	47.9	3.90
